# Comprehensive secretome profiling and CRISPR screen identifies SFRP1 as a key inhibitor of epidermal progenitor proliferation

**DOI:** 10.1038/s41419-025-07691-0

**Published:** 2025-05-03

**Authors:** Binbin Cheng, Shaohong Isaac Peng, Yunlong Y. Jia, Elton Tong, Scott X. Atwood, Bryan K. Sun

**Affiliations:** 1 Department of Dermatology, University of California Irvine, Irvine, CA USA; 2 Department of Developmental and Cell Biology, University of California Irvine, Irvine, CA USA

**Keywords:** Skin stem cells, Genomics

## Abstract

Secreted proteins are crucial for the structure and functions of the human epidermis, but the full repertoire of the keratinocyte secretome has not been experimentally defined. In this study, we performed mass spectrometry on conditioned media from primary human keratinocytes, identifying 406 proteins with diverse roles in adhesion, migration, proliferation, proteolysis, signal transduction, and innate immunity. To leverage this new dataset, we developed a novel colony formation assay-based CRISPR screen to investigate the functions of uncharacterized secreted proteins on epidermal stem cells. The screen identified six candidate proteins that promoted proliferation of epidermal progenitors and two proteins that inhibited it. Secreted frizzled-related protein-1 (SFRP1) was the most potent inhibitor. We discovered that SFRP1 restrained clonogenic keratinocyte proliferation by inhibiting Wnt signaling as well as blocking ectopic expression of leukemia inhibitory factor (LIF). Collectively, our study expands our knowledge of the keratinocyte secretome, establishes a novel CRISPR screen to assess the function of non-cell autonomous factors, and highlights SFRP1’s role in regulating epidermal balance.

## Introduction

The epidermis, the outermost aspect of the skin, is a stratified epithelium maintained by a balance between stem cell renewal, differentiation, and shedding from the outermost surface. Disruption of this equilibrium causes skin conditions that affect over 20% of the population, significantly impacting human health [1–3]. The epidermis faces ongoing challenges from injury, ultraviolet irradiation, microbes, and other environmental stressors [4–6]. While understanding the molecular mechanisms that maintain epidermal balance is crucial for treating skin diseases, these mechanisms are not fully known.

Secreted proteins play a major role in these processes. They orchestrate communication between cells, construct the extracellular matrix, and promote skin defense [7–9]. Determining the identity and functions of secreted proteins holds great potential to advance our understanding of skin health and disease. Epidermal stem/progenitor cells reside in the innermost basal layer and progressively differentiate as they move superficially towards the skin’s surface. Secreted proteins are critical to regulating epidermal stem cell decisions: Autocrine Wnt signaling promotes self-renewal [10] while epidermal growth factor (EGF) and transforming growth factor (TGF) family members can both stimulate proliferation and/or differentiation [11]. Beyond cell fate determination, secreted epidermal proteins are also mediators of skin inflammation. These include cytokines (e.g., IL-8, IL-6, tumor necrosis factor), growth factors (e.g., VEGF, EGF, GM-CSF, and TGF-β), and antimicrobial peptides (e.g., human β-defensin 2, LL-37, S100) [12–14]. Due to their extracellular location, secreted proteins have unique potential as biomarkers and drug targets, or as therapeutic molecules themselves [15, 16].

While ~80 secreted epidermal keratinocyte proteins have been identified to date [12, 17], the comprehensive epidermal keratinocyte secretome has not been experimentally defined, and the scope of its functions is incompletely understood. To address this knowledge gap, we performed mass spectrometry on conditioned media of undifferentiated and differentiated primary human keratinocytes to catalog the epidermal keratinocyte secretome. We determined a repertoire of 406 proteins, including over 100 proteins whose functional roles in the skin had not been studied in depth. To evaluate the potential roles of these secreted factors, we developed a colony formation-based CRISPR knockout screen capable of identifying non-cell autonomous factors regulating the renewal of epidermal progenitors. Through a combination of genetic depletion, recombinant protein rescue, RNA-seq, and ATAC-seq analyses, we took advantage of this new protein dataset to identify secreted Frizzled-related protein 1 (SFRP1) as a key regulator of human keratinocyte renewal.

## Results

### Defining the human keratinocyte secretome

**Fig. 1:**
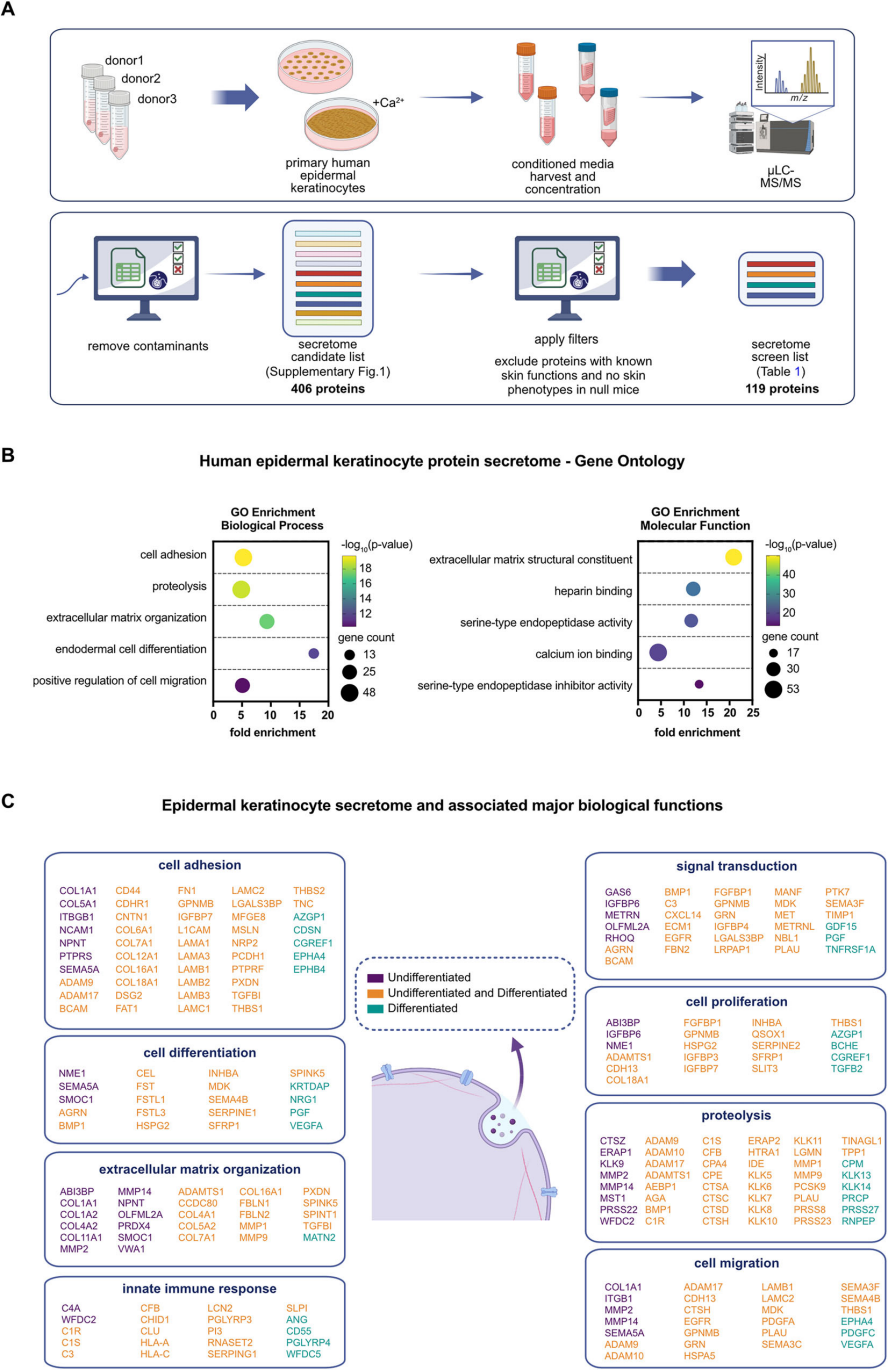
Profiling of the human epidermal keratinocyte secretome. **A** Schematic diagram of experimental approach. Mass spectrometry analysis was performed on conditioned media from primary human keratinocytes cultured under proliferation and differentiation conditions. **B** Gene Ontology (GO) enrichment analysis of keratinocyte secretome proteins. **C** Major biological functions and associated secreted proteins of human keratinocytes. Proteins were assigned to biological processes by GO enrichment. Biological processes with 20 or more associated proteins are shown.

To identify the proteins secreted by keratinocytes, we performed mass spectrometry on conditioned media collected from primary human neonatal keratinocytes (Fig. 1A). Keratinocytes were pooled from three unrelated individuals and propagated in defined keratinocyte culture medium under low calcium, low-density growth conditions (“undifferentiated”), as well as at elevated calcium (1.2 mM) and full confluence—conditions that induce keratinocyte differentiation (“differentiated”). Differentiation was confirmed by assessment of characteristic progenitor and differentiation-associated gene expression (Supplementary Fig. 1A).

Undifferentiated and differentiated keratinocyte conditioned media were collected and concentrated by ultrafiltration. Equivalent total protein from concentrated supernatants was resolved by SDS-PAGE electrophoresis, visualized by Coomassie Blue staining, and divided into gel sections to facilitate high-depth peptide identification. Each gel section was subjected to microcapillary reverse-phase HPLC coupled to tandem mass spectrometry (µLC-MS/MS) on an LTQ-Orbitrap mass spectrometer. µLC/MS-MS generated 18,175 and 17,267 spectral counts from undifferentiated and differentiated supernatants, respectively.

After correlation of spectra sequences to proteins, probable contaminants were removed (Supplementary Fig. 1B), and the list was filtered through the Human Protein Atlas database of secreted proteins to exclude abundant intracellular proteins that were likely to have been released into the supernatant from cell lysis. The final filtered set consisted of 406 proteins, which we propose as the human keratinocyte secretome (Supplementary Fig. 1C).

### Characteristics of the keratinocyte secretome

Keratinocyte secretome proteins participate in a broad array of biological functions. Gene Ontology (GO) enrichment analysis demonstrated substantial representation of secreted factors in the processes of cell adhesion, proliferation, migration, proteolysis, extracellular matrix remodeling, innate immunity, and signal transduction (Fig. 1B, C). Top molecular function GO terms included extracellular matrix, heparin binding, and serine-type endopeptidases and inhibitors. Comparison of the secretome of undifferentiated and differentiated keratinocytes revealed overlapping but distinct profiles, reflecting the dynamic changes in secreted proteins associated with epidermal differentiation. Several secreted proteins involved in extracellular matrix generation (e.g., collagens) were only detected in undifferentiated keratinocytes, while others involved with antimicrobial defense were represented only in differentiated keratinocytes (Fig. 1C). Overall, these results expand the known repertoire of experimentally determined keratinocyte-secreted proteins and underscore their involvement in vital elements of skin biology.

Of the 406 proteins of the secretome list, we identified 119 proteins that had experimental association with a skin phenotype in mice (Mouse Genome Informatics database, https://www.informatics.jax.org). This included proteins for which mutations in cognate genes cause human skin diseases, such as collagen 7A1 and epidermolysis bullosa (Online Inheritance in Man/OMIM 120120); serine protease inhibitor Kazal type 5 and Netherton syndrome (OMIM 605010); and secreted LY6/Plaur domain-containing protein-1 and Maleda syndrome (OMIM 606119). Of the remaining proteins in the secretome, 168 proteins had no skin phenotype in mouse gene knockout studies. Excluding proteins with known phenotypes and putatively negative phenotypes, a remaining list of 119 proteins had either not been characterized extensively or had not been studied at all (Table 1).

**Table 1:** Keratinocyte-secreted proteins targeted in the CRISPR screen.

A2ML1	CPB2	FCRL5	ITIH1	LTBP1	PAM	QPCT	SHBG
ADAM10	CPM	FKBP2	ITIH2	LTBP2	PAPPA2	RCN2	SIAE
ADAM9	CRELD1	GALNT2	ITIH3	LTBP4	PCDH1	RHOQ	SLPI
AKR1C1	CUTA	GALNT6	KLK10	LYPD2	PDDC1	RNASET2	SLURP1
ALDOA	CYR61	GANAB	KLK11	MAMDC2	PDIA3	RNPEP	SPINT2
APOB	DKK3	GGH	KLK13	MDK	PDIA6	RPLP2	SULF2
APOH	DMKN	GLG1	KLK9	METRN	PI3	SAA4	TCN2
ATP6AP1	DNASE2	GOT2	KRTDAP	METRNL	PIK3IP1	SBSN	TINAGL1
B3GAT3	DSG2	HS3ST1	LAMA5	MXRA5	PLBD2	SDF4	TWSG1
B4GALT4	ECM1	HSP90B1	LAMB1	NME3	PLOD2	SEMA3C	VSTM2L
B4GALT5	EPS8L1	HSPA13	LAMB2	NOMO3	PPIC	SEMA3F	WFDC2
C21orf33	ERAP2	IGFBP6	LAMC1	NT5E	PRSS22	SEMA5A	WFDC5
CALU	ERP29	IGFL1	LFNG	NUCB1	PRSS23	SERPINA1	WNT5A
CHI3L1	FAT1	IGFL3	LMAN2	OAF	PSAPL1	SERPINA4	XYLT1
CNPY2	FBLN2	IPO9	LRPAP1	OLFML2A	PSMD1	SFRP1	

Many of the uncharacterized secreted proteins had biologically plausible mechanisms that could impact epidermal function. These included proteins regulating signal transduction pathways (DKK3, EPS8L1, SFRP1), proteases and protease inhibitors (KLK9, KLK10, SLPI), metabolic enzymes (ALDOA, AKR1C1), and cell differentiation-associated factors (SBSN, KRTDAP). Because of the large number of candidates, we decided to perform a functional screen to test their potential biological roles.

### A colony formation assay-based secretome CRISPR screen

We aimed to identify keratinocyte secretome proteins that had roles in epidermal progenitor self-renewal, because this biological process is essential to skin homeostasis and is disrupted in many skin diseases [18–21]. We reasoned that a screening approach would have the benefit of highlighting protein regulators that had strong and non-redundant regulatory roles and would benchmark effect sizes of candidates relative to one another.

**Fig. 2:**
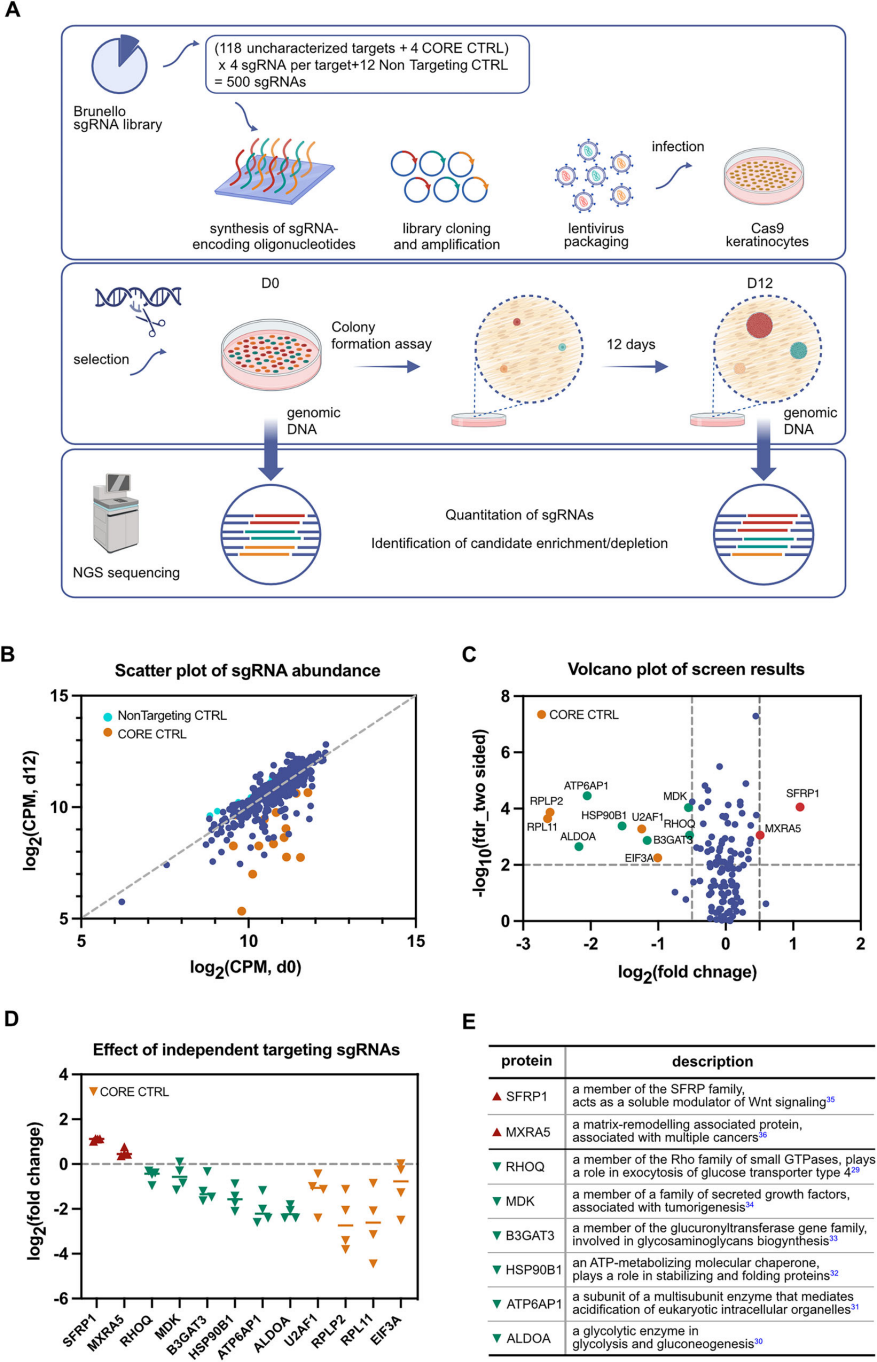
A colony formation assay-based secretome CRISPR screen identifies regulators of epidermal stemness. **A** Schematic diagram of colony formation-based secretome CRISPR screen. The CRISPR lentiviral library was transduced into Cas9-expressing keratinocytes and seeded onto a fibroblast feeder layer for colony formation. Genomic DNA was harvested at initial and final timepoints and sequenced to quantitate sgRNA abundance. **B** Scatter plot of normalized sgRNA abundance. Results were averaged from two experimental replicates. Nontargeting control (CTRL) sgRNAs are shown in cyan. Positive control core essential gene controls (CORE CTRL) are shown in orange. **C** Volcano plot of screen results. A discovery threshold for a positive hit was defined as | log_2_(fold change)| > 0.5 and false discovery rate < 0.01. Core controls are labeled in orange. Candidate hits from sgRNA depletion are in teal. Candidate hits showing sgRNA enrichment hits are in red. **D** SgRNA abundance of individual targeting sgRNAs. Each secreted protein candidate in the screen was targeted by four independent sgRNAs. Abundance changes of each sgRNA are shown, averaged between two experimental replicates. **E** Identity and described functions of eight candidate protein hits from the CRISPR screen.

To assay epidermal progenitor stem cell potential, we developed a colony formation-based CRISPR screen (Fig. 2A). The screen takes advantage of an assay in which keratinocytes are seeded sparsely as single cells onto a fibroblast feeder layer. Seeded keratinocytes develop into colonies with different clonogenic capabilities [22, 23]. This assay design would allow for sequencing-based quantitation of proliferative potential while taking advantage of the distance between seeded colonies to reduce non cell autonomous rescue [24–26].

We generated a single guide RNA (sgRNA) library composed of four independent sgRNAs targeting each of the candidate 119 proteins. The sgRNA guide sequences were taken from a validated Brunello sgRNA human CRISPR library [27]. As positive controls, we included sgRNAs to three core essential genes (EIF3A, RPL11, U2AF1), which are genes that are necessary for cell survival across a range of conditions and cell types [28]. After generation of the library, we recognized that one of the secreted candidates, RPLP2, was also a core essential gene. We also included 12 non-targeting controls. In total, the lentiviral sgRNA library contained 500 sgRNAs.

We transduced the sgRNA library into Cas9-expressing keratinocyte cell line (Supplementary Fig. 2A–D) at a multiplicity of infection of 0.16. A total of 2.5 × 10^5^ transduced and selected keratinocytes, representing 500x library coverage, were seeded and propagated for 12 days (Supplementary Fig. 2E). Genomic DNA was collected at the initial timepoint and after 12 days of colony cultivation. SgRNA representation was quantified by next-generation sequencing (Fig. 2B). The screen was performed twice.

### CRISPR screen identifies SFRP1 as a key regulator of epidermal stemness

NGS confirmed 99.8% coverage of the 500 sgRNAs and 100% coverage of the 123 secretome targets of the library in all samples (Supplementary Fig. 2F). Quantification of sgRNA representation showed high technical reproducibility (Supplementary Fig. 2G, H). SgRNAs targeting the four core essential genes were under-represented in the post-assay timepoint (Fig. 2B, C), confirming that the CRISPR-based knockout was effective and that the screen was sensitive to detect functional genes. The 12 non-targeting sgRNAs showed non-significant changes between day 0 and day 12, indicating that they were neutral to assay readout.

We applied a threshold of |log_2_ (fold change)| > 0.5 and a false discovery rate of <0.01 to define sgRNA screen hits. Based on these thresholds, eight secretome proteins were identified as screen hits (Fig. 2C). The four independent sgRNAs for each of the 8 candidate hits showed concordance in the directionality of their effect (Fig. 2D). Six screen hits (RHOQ, MDK, B3GAT3, HSP90B1, ATP6AP1, and ALDOA) [29–34] were identified by under-represented sgRNAs. Two candidates (SFRP1 and MXRA5) [35, 36] were identified by over-represented sgRNAs (Fig. 2E).

### SFRP1 restrains keratinocyte progenitor proliferation

We focused on the two candidates whose sgRNAs were enriched in the screen, because this implies that their normal function may be to inhibit stem cell proliferation. The candidate with the lower FDR (0.00009) and higher fold change (log_2_FC = 1.102) was secreted Frizzled related protein-1 (SFRP1). SFRP1 is a member of a larger SFRP family [37] that regulates Wnt signaling. Prior studies demonstrated that SFRP1 mediates activation, proliferation, and differentiation of hair follicle stem cells [38–40]. Promoter methylation and reduced expression of SFRP1 are observed in keloids and cutaneous squamous cell carcinoma [41, 42]. Furthermore, Sfrp1 depletion in a mouse skin carcinogenesis model promoted early tumor initiation and tumorigenic potential [43]. Together, these findings supported a role for SFRP1 in different facets of skin biology and disease. However, the function of SFRP1 on human interfollicular epidermal keratinocyte self-renewal has not been described.

**Fig. 3:**
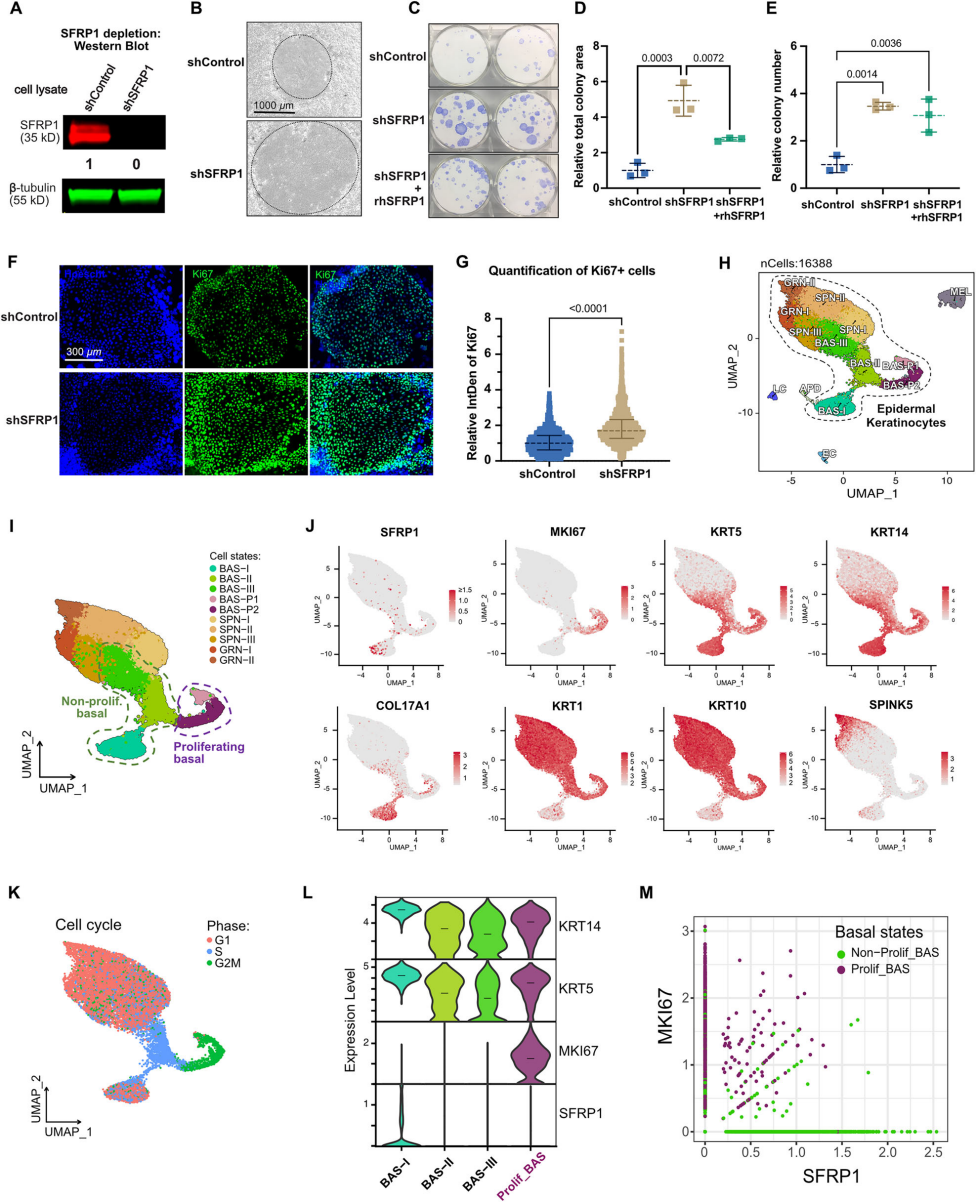
SFRP1 restrains keratinocyte progenitor proliferation. **A** Immunoblot of SFRP1 protein depletion in control (shControl) and SFRP1-depleted (shSFRP1) keratinocytes. Normalized SFRP1 relative expression is denoted. sgControl was set to 1 for comparation. **B** Colony morphology of shControl and shSFRP1 keratinocytes at endpoint of colony-formation assay. Scale bar, 1000 µm. **c** Crystal violet staining of colonies from shControl, shSFRP1, and shSFRP1 with recombinant human SFRP1 protein (rhSFRP1). Recombinant SFRP1 protein was applied to concentration of 1 ng/ml. **D** Quantification of colony formation area in (**C**). The average of shControl replicates was set to 1. Data are means ± SD (*n* = 3, one-way ANOVA with a Tukey’s honestly significant differences [HSD] post hoc test). *p* values are labeled above individual comparisons. **E** Quantification of colony formation number in (**C**). The average of shControl replicates was set to 1. Data are means ± SD (*n* = 3, one-way ANOVA with a Tukey’s honestly significant differences [HSD] post hoc test). **F** Ki67 immunofluorescence of control and SFRP1 knockdown keratinocyte colonies. Ki-67+ is labeled in green, and nuclei stained in blue. Scale bar, 300 µm. **G** Quantification of Ki-67+ cells in (**F**). Each datapoint represents the relative integrated density of Ki-67 signal. The median of shControl was set 1 for comparison. Data are median with interquartile range. (*n* = 862 and 1378 for shControl and shSFRP1, respectively, two-tailed unpaired student’s *t*-test). **H** UMAP visualization of all cell types or states identified in the human neonatal skin epidermis scRNA-seq dataset (*n* = 5). BAS basal keratinocytes, SPN spinous keratinocytes, GRN granular keratinocytes, APD skin appendage-related cells, MEL melanocytes, EC endothelial cells, LC Langerhans cells. **I** UMAP visualization of scRNA-seq data from human neonatal skin keratinocytes, colored by different cell states. These keratinocytes represent a subset derived from the neonatal foreskin epidermis UMAP in (**H**). Both non-proliferating (green dashed line) and proliferating (purple dashed line) basal keratinocytes are highlighted on the UMAP. BAS basal keratinocytes, SPN spinous keratinocytes, GRN granular keratinocytes. **J** Top: scaled and normalized expression of SFRP1, dividing cell marker, and skin basal keratinocyte marker genes; Bottom: feature plots for canonical markers of skin keratinocytes. **K** UMAP projection colored by cell-cycle phase. **L** Violin plot depicting the expression distribution of genes across different basal cell states. Horizontal bars within the violin plots indicate median values. **M** Scatter plot showing the co-expression analysis of MKI67 and SFRP1. Each point represents a single cell.

To confirm the results of the screen, we verified that the four SFRP1-targeting sgRNAs depleted protein expression of SFRP1 as intended (Supplementary Fig. 3A). The effect of all four independent sgRNAs was concordant (Fig. 2D), and the phenotype of increased clonogenicity was repeated using individual knockouts of a subset of the screening sgRNAs (Supplementary Fig. 3B–D). To further corroborate the phenotype and rule out effects of Cas9 expression on epidermal keratinocyte proliferative potential, we performed the colony formation assay using short hairpin/RNA interference targeting of SFRP1 (shSFRP1) in primary keratinocytes (Fig. 3A and Supplementary Fig. 3E). Consistent with the results from CRISPR knockout, we observed that shSFRP1 displayed an increased number and size of keratinocyte colonies compared to control (Fig. 3B–E). To assess if larger colony size was associated with increased proliferation and/or decreased cell apoptosis, we performed immunostaining for the proliferation marker Ki-67 and the apoptosis marker cleaved caspase-3. We observed low and equivalent caspase-3 signal in keratinocyte colonies of both shControl and shSFRP1 (Supplementary Fig. 3F). However, we observed enhanced Ki-67 positivity in shSFRP1 colonies, which were accentuated in the expanding peripheral regions of the colonies (Fig. 3F, G). Together, these results indicated that SFRP1 depletion enhances cell proliferation.

To confirm the specificity of the phenotype to SFRP1, we treated shSFRP1 knockdown cells with recombinant human SFRP1 protein (rhSFRP1, Fig. 3C–E). Application of exogenous rhSFRP1 reduced the clonogenicity of shSFRP1 keratinocytes towards levels of the control cells, showing that the knockdown phenotype was the result of depleting extracellular SFRP1. The results from the screen, confirmatory shRNA experiments, and exogenous rescue indicated that SFRP1 functions to inhibit epidermal progenitor self-renewal, and its depletion leads to increased epidermal proliferation.

To assess the spatial expression of SFRP1 within the interfollicular epidermis, we analyzed single-cell RNA sequencing (scRNA-seq) data from human neonatal skin (Fig. 3H). Clustering delineated keratinocyte subpopulations: basal keratinocytes (BAS), characterized by high expression of KRT5, KRT14, and COL17A1; spinous keratinocytes (SPN), marked by KRT1 and KRT10; and granular keratinocytes (GRN), identified by SPINK5 among other markers. Additional subclustering further resolved distinct subpopulations within BAS, SPN, and GRN layers. Overall, SFRP1 expression was most highly represented in basal cells compared with the spinous and granular populations (Fig. 3H, J), mirroring its mRNA and protein expression in primary cultured keratinocytes, where levels were highest in undifferentiated cells and progressively decreased with calcium-induced differentiation (Supplementary Fig. 3G–I).

SFRP1 has been recognized in two isoforms (Ensembl IDs: SFRP1_201 and SFRP1_202). Using quantitative RT-PCR with isoform-specific primers, we determined that SFRP1_201 is the predominantly expressed isoform in human keratinocytes, while SFRP1_202 was undetectable.

Basal epidermal keratinocytes can be further subclassified into non-proliferating (BAS-I, BAS-II, BAS-III) and proliferating subpopulations (BAS_P1, BAS_P2), distinguished by differential MKI67 expression (Fig. 3H–J) and cell cycle phase (Fig. 3K). Within these subpopulations, proliferating basal cells did not express SFRP1, whereas cells with high SFRP1 expression were predominantly non-proliferative (Fig. 3J–L). A co-expression analysis further demonstrated that MKI67 and SFRP1 expression in basal epidermal keratinocytes are predominantly mutually exclusive (Fig. 3M). Collectively, these data suggest that SFRP1 is preferentially expressed in a quiescent subpopulation of basal keratinocytes and that its repression can activate proliferation.

### SFRP1 knockdown in human epidermal organoids

**Fig. 4:**
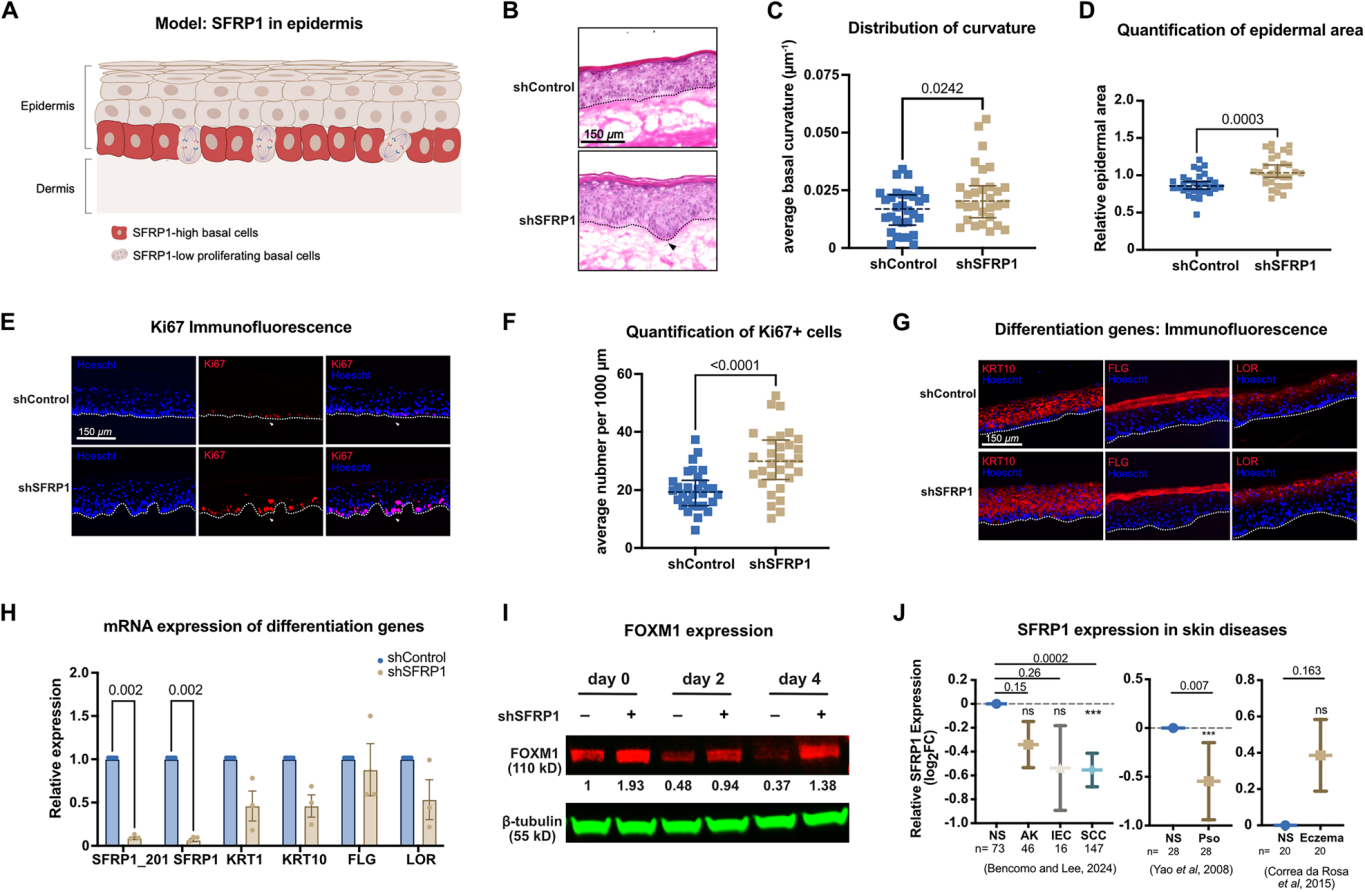
SFRP1 knockdown in human epidermal organoids. **A** Schematic diagram of a working model of SFRP1 in the interfollicular epidermis. **B** Hematoxylin and eosin (H&E) staining on control and SFRP1-depleted epidermal organotypic tissues. Dotted black lines denote the basement membrane. Black arrowheads highlight example regions of collections of progenitor keratinocytes. Scale bar, 150 µm. **C** Quantification of basal curvature in (**B**). Each datapoint represents the curvature measurement of the basal layer of a sampled H&E image. Data are median ± interquartile range. (*n* = 30 from three independent biological replicates, two-tailed unpaired student’s *t*-test). **D** Quantification of epidermal area in (**B**). Each datapoint represents the relative area measurement of the epidermis of a sampled H&E image. Data are median ± interquartile range. (*n* = 30 from three independent biological replicates, two-tailed unpaired student’s *t*-test). **E** Immunofluorescence of epidermal organotypic tissues. Ki-67 marks proliferating keratinocytes. White dotted lines demarcate the basement membrane. Scale bar, 150 µm. **F** Quantification of Ki-67 positive cells in (**E**). Each datapoint represents the count of Ki-67+ cells from a sampled image, normalized to tissue length. Data are median ± interquartile range. (*n* = 30 from three biological replicates, evaluated with two-tailed unpaired student’s *t*-test). **G** Immunofluorescence of keratin 10, filaggrin, and loricrin in control and SFRP1-depleted epidermal organotypic tissues. Dotted white lines denote the basement membrane. Scale bar, 150 µm. **H** Quantitative RT-PCR of SFRP1 (isoform SFRP1_201, and total SFRP1) and differentiation-associated genes (KRT1, KRT10, FLG, LOR) in organoid epidermis in control and SFRP1 depleted tissues. **I** Immunoblot of FOXM1 in shControl and shSFRP1 primary keratinocytes over time course of in vitro differentiation. For relative quantitation, FOXM1 signal was normalized to beta tubulin, and the shControl day 0 signal was set to 1. **J** SFRP1 mRNA expression in skin diseases. Gene expression data from studies of keratinocyte cancers, psoriasis, and eczema were compiled. Selected studies included patient-matched normal skin (NS). Two-sided *t*-tests were performed to compare expression in NS vs. disease states. Data are mean ± SEM. Sample sizes (*n*) for each group depicted in figure. NS normal skin, AK actinic keratosis, IEC intraepidermal carcinoma, SCC squamous cell cancer, Pso psoriasis. *P* values shown above pairwise comparisons.

Based on the screening, perturbation, and expression results, we generated a working depiction of SFRP1 expression and relationship to basal proliferation within the interfollicular epidermis (Fig. 4A). To test this relationship in a more physiological context, we evaluated the effect of SFRP1 depletion in epidermal organoids, in which primary keratinocytes reside in a more native three-dimensional environment. Control vs. shSFRP1 keratinocytes were seeded onto the basement membrane of devitalized, acellular human dermis and cultivated at an air-liquid interface for one week to induce epidermal stratification (Fig. 4B).

SFRP1-depleted epidermal tissue displayed a phenotype characterized by hypercellular collections of keratinocytes in basal epidermal layers. By quantitating curvature of the basement membrane, we found that SFRP1-depleted epidermis resulted in greater undulations of the basement membrane compared to control (Fig. 4C). The histology of the more superficial epidermal layers was grossly unaffected. To quantitate overall effects on epidermal thickness, we compared the overall area of the epidermis per visual field between shControl vs shSFRP1 epidermis, using the basement membrane and upper stratum granulosum as lower and upper boundaries. We found an 18% increase in epidermal area in shSFRP1, indicating that the average overall epidermal thickness was increased by SFRP1 depletion, although the magnitude of the impact on thickness was modest (Fig. 4D).

Next, we performed MKI67 immunostaining to visualize cell proliferation. In control tissue, MKI67+ cells decorated a subset of cells along the basal layer of the epidermis (Fig. 4E, top row). In contrast, shSFRP1 epidermal tissue displayed markedly increased MKI67+ staining, with clustering of MKI67+ cells at hypercellular regions within and adjacent to the basal layer (Fig. 4E, F). We then assessed whether SFRP1 depletion affected epidermal differentiation by performing immunofluorescence staining for differentiation-associated proteins keratin 10, filaggrin, and loricrin. Both qualitative and quantitative analyses revealed no statistically significant differences in the abundance or distribution of these proteins (Fig. 4G). In addition, quantitative RT-PCR showed no statistically significant changes in mRNA levels of differentiation markers, although there was a trend toward reduced expression in shSFRP1 tissue (Fig. 4H). We reasoned that this trend may not reflect an intrinsic effect on blocking differentiation but was more likely an averaging effect due to an expanded population of proliferative basal progenitors. This interpretation was supported by a time-course experiments of calcium-induced differentiation in vitro, where increased FOXM1 expression—a marker of epidermal proliferation—was sustained even after prolonged differentiation stimulus (Fig. 4I), indicating the strong effect of SFRP1 depletion to promote proliferation.

Excessive epidermal keratinocyte proliferation is a hallmark of skin diseases such as psoriasis and keratinocyte cancers. To examine if SFRP1 expression is altered in patients with these conditions (Fig. 4J), we evaluated RNA-seq and microarray datasets in the NIH Gene Expression Omnibus (NIH GEO). SFRP1 expression was decreased in squamous cell skin cancer (SCC) compared to normal control skin [44]. In psoriasis, a skin condition typified by hyperproliferative epidermis, SFRP1 expression was also significantly lower than control normal skin [45]. By contrast, in eczema/atopic dermatitis, there was no significant difference in SFRP1 expression between control and diseased tissue [46]. These data showed that SFRP1 expression is reduced in hyperproliferative skin diseases such as SCC and psoriasis but not in eczema.

### SFRP1 constrains LIF expression in addition to Wnt signaling

**Fig. 5:**
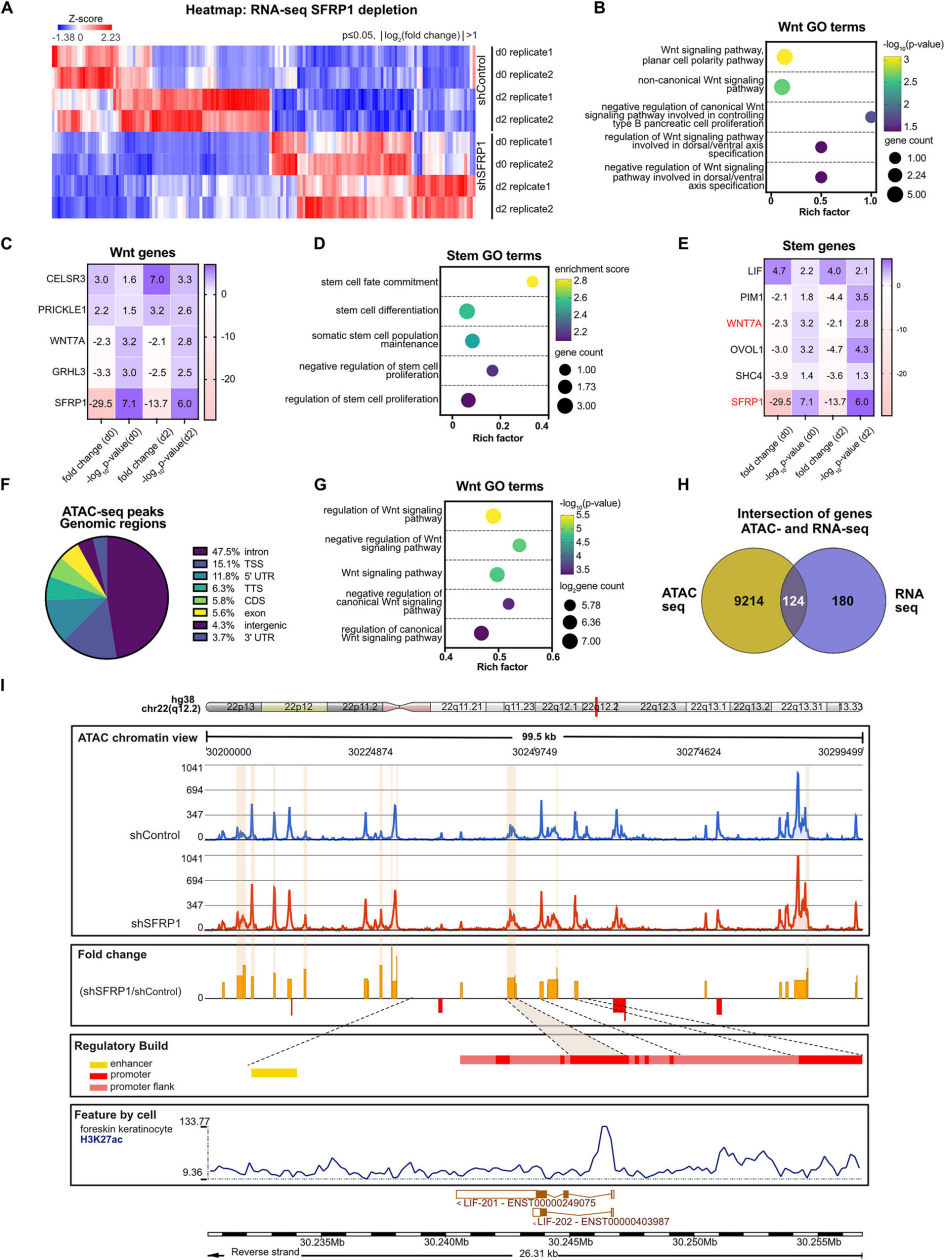
SFRP1 inhibits Wnt signaling and other stemness regulators. **A** Heatmap of differential gene expression in SFRP1-depleted keratinocytes. Differentially expressed genes (DEGs) between SFRP1-depleted and control keratinocytes were identified on day 0 and day 2 timepoints. DEGs were defined by threshold of *p* value ≤ 0.05 and | log_2_ (fold change)| > 1. **B** Top enriched GO terms (ranked by p-value) related to Wnt signaling. **C** Heatmap of gene expression for representative genes in (**B**). **D** Top enriched GO terms (ranked by *p* value) related to stem cell regulation. **E** Heatmap of gene expression for representative genes in (**D**). Genes associated with Wnt signaling are labeled in red. **F** Distribution of SFRP1-associated differential ATAC-seq peaks across genomic regions. Differential peaks were defined by threshold *p* value ≤ 0.05 and |fold change| > 1.5. **G** Top enriched Wnt GO (ranked by *p* value) of differential ATAC-seq associated genes. **H** Venn diagram of DEGs in RNA-seq and differential peak-associated genes in ATAC-seq. **I** Chromatin accessibility plot at the LIF genomic locus (chr22:30, 240, 453- 30, 246, 759). Shadowed fold change peaks meet threshold of >1.5 (shSFRP1 vs. shControl). Tracks were aligned with Ensembl LIF regulatory build and H3K27ac ChIP-seq data from foreskin keratinocytes.

To determine the downstream transcriptional impacts of SFRP1, we performed RNA sequencing of SFRP1-depleted cells in undifferentiated (day 0) and early differentiated (day 2) keratinocytes. The shSFRP1 transcriptome was compared to shControl at each timepoint, and differentially expressed genes (DEGs) were classified as those with *p* value ≤ 0.05 and |log_2_(fold change)| > 1. Combining results from both timepoints, we identified 304 DEGs associated with SFRP1 depletion (Fig. 5A).

GO analysis of DEGs was headlined by terms related to keratinization, endopeptidase activity, and calcium channel regulator activity (Supplementary Fig. 4A). Consistent with its role in regulating Wnt, GO terms related to canonical and non-canonical Wnt pathways were enriched (Fig. 5B). Genes comprising these GO terms included WNT7A, WNT3A, WNT6, and DRD2 [47], most of which showed increased expression with SFRP1 depletion (Fig. 5C, and Supplementary Fig. 4B, C). Because SFRP1 is an inhibitor of Wnt signaling, upregulation of Wnt-activation related transcripts in shSFRP1 cells was consistent its established function.

Beyond effects on transcription of Wnt-related genes, we also assessed more broadly how SFRP1 affected expression of gene transcripts associated with stem cell fate. Examining the genes that comprised enriched GO terms related to stem cell regulation (Fig. 5D, E, and Supplementary Fig. 4D, E, Supplementary Tables 5, 6) [48, 49], we found enrichment of well-defined stem cell regulators including the transcription factor TP53 [50], NOTCH1 [51], and SMO [52], reflecting an impact of SFRP1 on major signaling pathways that control epidermal progenitor stemness.

To gain insight into the regulatory mechanisms underlying the transcriptome changes, we performed assay for transposase accessible chromatin sequencing (ATAC-seq), comparing shSFRP1 to shControl keratinocytes. We applied a threshold of *p* value ≤ 0.05 and |fold change| > 1.5 to define 19,136 differential ATAC peaks. The peaks mapped to introns, intergenic spaces, and promoters (Fig. 5F).

We assigned peaks to their nearest annotated genes and performed GO analysis of differential peak-associated genes. We found enrichment of terms related to signal transduction, cell movement, and anatomical structure development (Supplementary Fig. 4G). Wnt-related GO terms were represented (Fig. 5G), consistent with SFRP1 causing genomic regulatory changes to control expression of Wnt-related genes. To further integrate the two datasets, we performed a correlation analysis between the genes identified in the RNA-seq and ATAC-seq results (Fig. 5H). The gene set intersection between these two experiments contained 124 genes. One gene within this intersection that drew particular attention was leukemia inhibitory factor (LIF). In the RNA-seq dataset, LIF exhibited the greatest increase in expression among DEGs associated with stem-related terms (Fig. 5E). The chromatin accessibility landscape at the LIF genomic locus showed increased accessibility at the LIF promoter upon SFRP1 depletion (Fig. 5I). To the best of our knowledge, however, LIF had not been connected to canonical Wnt signaling, so we chose to further explore if this candidate played a role in the SFRP1 knockout phenotype in epidermal keratinocytes.

### LIF promotes epidermal progenitor renewal and is inhibited by SFRP1

LIF is a secreted, pleiotropic cytokine expressed in a broad range of tissues and affects cell growth, differentiation, and inflammation. In the skin, its overexpression has been associated with development of hyperplastic epidermis [53] and epidermal tumors [54], but its function in the context of other stemness regulators and the mechanisms that control its expression in the skin are unknown.

**Fig. 6:**
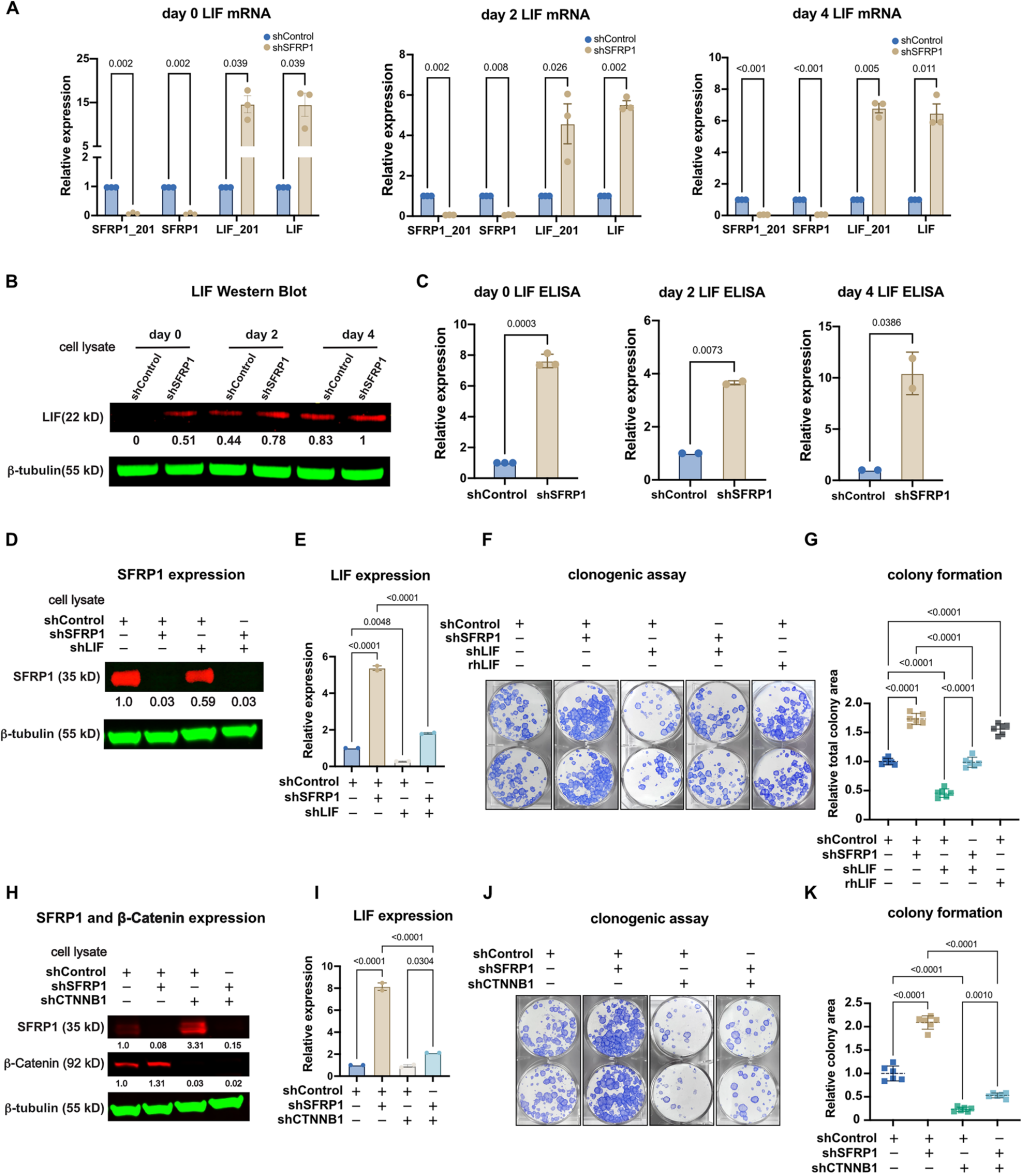
LIF promotes epidermal progenitor renewal and is inhibited by SFRP1. **A** Quantitative RT-PCR of SFRP1 and LIF mRNA in control vs. SFRP1-depleted keratinocytes across an in vitro differentiation time course (day 0-2-4). SFRP1_201, isoform SFRP1_201; SFRP1, total SFRP1; LIF_201, isoform LIF_201; LIF, total isoforms. Data are mean ± SEM. (*n* = 4, multiple paired student’s *t*-test). *p* values denoted above comparisons. **B** Immunoblot of LIF protein in control vs SFRP1-depleted primary keratinocytes across an in vitro differentiation time course. Relative intensity of LIF is denoted, using shSFRP1 at day 4 = 1.0 for comparison. **C** ELISA of LIF in control vs. SFRP1-depleted primary keratinocytes. shControl was set to 1 for comparison. Data are mean ± SEM. (*n* = 2, two-tailed ratio student’s *t*-test). **D** Immunoblot of SFRP1 in primary keratinocytes following control, SFRP1 knockdown (shSFRP1), and/or LIF knockdown (shLIF). Relative intensity of SFRP1 is denoted and normalized to beta tubulin. sgControl was set to 1 for comparison. **E** ELISA of LIF in primary keratinocytes following control, shSFRP1, and/or shLIF. Data are mean ± SD. shControl was set to 1 for comparison. (*n* = 3, one-way ANOVA with a Tukey’s HSD post hoc test). **F** Crystal violet staining of keratinocyte colonies generated following treatment with shControl, shSFRP1, shLIF, and/or recombinant human LIF (rhLIF). **G** Quantification of colony formation in (**F**). ShControl was set to 1 for comparison. Data are means ± SD. (*n* = 6, one-way ANOVA with a Tukey’s HSD post hoc test). **H** Immunoblot of primary keratinocyte protein lysates following treatment with shControl, shSFRP1, and/or ß-catenin knockdown (shCTNNB1). Relative intensity of SFRP1 and CTNNB1 is denoted and normalized to beta tubulin. shControl was set to 1 for comparison. **I** ELISA of LIF in primary keratinocytes following control, shSFRP1, and/or shCTNNB1. Data are mean ± SD. shControl was set to 1 for comparison. (*n* = 2, one way ANOVA with Tukey’s HSD). **J** Crystal violet staining of keratinocyte colonies generated following treatment with shControl, shSFRP1, and/or shCTNNB1. **K** Quantification of colony formation in (**J**). ShControl was set to 1 for comparison. Data are means ± SD. (*n* = 6, one-way ANOVA with a Tukey’s HSD post hoc test).

We measured the RNA and protein expression of LIF in undifferentiated and differentiated epidermal keratinocytes using qRT-PCR, immunoblot, and ELISA. LIF expression was lowest in undifferentiated progenitors and increased modestly during in vitro differentiation (Supplementary Fig. 5A–D). In undifferentiated conditions of wild-type keratinocytes, LIF was near or below experimental limits of detection by qRT-PCR, immunoblot, and ELISA. LIF was not present in the unfiltered µLC/MS-MS based keratinocyte secretome list, further indicating that its expression in keratinocytes is low or absent. SFRP1 depletion in keratinocytes led to increased LIF mRNA and protein expression (Fig. 6A–C). Protein quantitation by immunoblot and ELISA showed relative and absolute upregulation compared to control. Together, the results from RNA and protein quantitation data indicated that LIF is expressed at a low or negligible level in undifferentiated epidermal keratinocytes but was ectopically induced by SFRP1 depletion.

We wanted to evaluate if ectopic LIF expression contributed to the increased proliferative potential phenotype seen with SFRP1 depletion. We performed a clonogenic assay comparing several conditions: SFRP1 knockdown, LIF knockdown, and double SFRP1 and LIF knockdown (Fig. 6D, E). As shown before, depletion of SFRP1 increased clonogenicity. However, dual knockdown of SFRP1 and LIF partially but incompletely blocked the increased clonogenic phenotype observed with SFRP1 knockdown (Fig. 6F, G, and Supplementary Fig. 5E). Recombinant human LIF protein (rhLIF), added directly to media in the clonogenicity assay, was sufficient to increase colony formation, supporting the effect of LIF in epidermal progenitors (Fig. 6F). Together, these results indicated that the effect of SFRP1 on epidermal stemness is mediated in part through a downstream induction of LIF expression.

The best characterized function of SFRP1 is its regulation of Wnt signaling. Activation of canonical Wnt signaling converges on stabilization of beta-catenin (CTNNB1), which cooperates with TCF/LEF transcription factors in the nucleus to activate target gene expression [55]. We sought to determine if the effect of SFRP1 on LIF expression and epidermal stemness could be attributed entirely to its role in suppressing Wnt signaling. To do so, we assessed LIF protein expression and clonogenic potential in control, SFRP1 knockdown, CTNNB1 knockdown, and double shSFRP1/shCTNNB1 knockdown (Fig. 6H). LIF was upregulated after SFRP1 knockdown, and this induction was partially blocked by concurrent CTNNB1 knockdown (Fig. 6I). Concordant with these results, the induction of colony formation by SFRP1 knockdown was partly inhibited by simultaneous CTNNB1 knockdown (Fig. 6J, K, and Supplementary Fig. 5F). In summary, these results indicated that SFRP1 impacts epidermal clonogenic potential largely through regulation of canonical Wnt signaling. However, the data also reveal a previously unrecognized contribution of SFRP1 to epidermal regulation through a canonical Wnt-independent mechanism, including suppression of LIF.

## Discussion

The lack of a systematic catalog of the human epidermal keratinocyte secretome has hindered progress in characterizing new candidates involved in epidermal homeostasis and identifying key regulatory proteins among them [12, 17]. In this study, we performed mass spectrometry on conditioned media from proliferating and differentiated keratinocytes, identifying 406 secreted proteins that constitute the human epidermal keratinocyte secretome. This work addresses the existing knowledge gap and provides an unbiased and more comprehensive profile of the keratinocyte secretome. Furthermore, by optimizing a screening strategy for secreted factors, we tested 119 proteins that have been either minimally characterized or not studied at all, offering new insights into skin biology.

Dissecting the functions of secreted proteins, which can act on distant cells, presents a unique challenge. Pooled knockout screens are ineffective because secreted factors from neighboring wild-type cells can mask knockout effects. Alternative screening approaches include gain-of-function strategies, such as individually applying recombinant protein candidates to test their effects, or overexpressing gene candidates in a cell type that does not ordinarily express those genes [56–62]. While productive, these approaches can be resource-intensive. To overcome this hurdle, we developed a colony formation-based CRISPR knockout screen specifically designed to identify secreted factors regulating epidermal progenitor stemness. Our CRISPR screen showed technical robustness and reproducibility. This approach successfully prioritized eight candidate regulators from a set of 119, highlighting its potential for parallel assessment of secreted protein functions.

Among these candidates, we identified SFRP1 as a potent extracellular inhibitor of epidermal progenitor proliferation. We demonstrate that epidermal SFRP1 functions through its established role of inhibiting Wnt signaling but also controls other regulators, such as LIF. Wnt signaling in keratinocytes is complex and context-dependent, with distinct ligands influencing proliferation or differentiation. For example, Wnt16 promotes proliferation, whereas Wnt5a enhances calcium-induced differentiation, both via B-catenin signaling [63, 64]. In our RNA-seq analysis, SFRP1 depletion downregulated Wnt7a, a ligand known to promote melanocyte differentiation via suppression of Notch signaling [65]. The multifaceted role of Wnt signaling underscores SFRP1’s potential to regulate critical aspects of skin biology, both within keratinocytes and through interactions with other cell types.

During the course of this research, the characterization of mouse Sfrp1 knockout (Sfrp1-ko) skin was reported, with a focus on its phenotype in hair follicle dynamics and propensity for skin carcinogenesis [38, 43]. Notably, in the hair follicle, Sfrp1-ko enhanced hair follicle stem cell proliferation and accelerated the hair follicle cycle. While the interfollicular epidermis was initially observed to be normal, later postnatal timepoints revealed an increase in Ki-67+ staining, suggesting that Sfrp1 could play a role in postnatal epidermal homeostasis. This observation raises the possibility of a distinct function for Sfrp1 in maintaining epidermal homeostasis after development, in contrast to its role in embryonic development. In contrast to the mouse model, our study uncovers the specific impact of SFRP1 depletion in human epidermis, providing new insights into its potential role in adult skin homeostasis and highlighting the translational relevance of our findings.

In addition to SFRP1, other candidate hits from the screen are attractive candidates to assess for roles in human epidermal biology. Midkine (MDK), another hit from the screen, is a growth factor cytokine that is induced upon wounding in a salamander regeneration model [66] and is essential for wound epidermis expansion after injury. In human skin, midkine overexpression is observed in keratinocyte cancers [67] and can stimulate keratinocyte proliferation in vitro [68]. In normal physiologic conditions it is expressed in basal epidermis, suggesting a potential role in progenitor/stem homeostasis. Another screen hit, matrix remodeling associated 5 (MXRA5), has also been associated with multiple cancers [36, 69]. When knocked out in our screen, it showed increased clonogenicity, indicating that it may function as a repressor of epidermal stemness. These and other candidates from the screen are promising candidates for further investigation.

We acknowledge that our experimental design has several limitations. Secreted proteins were identified from conditioned media of primary human keratinocytes in vitro, which may not fully recapitulate secreted proteins in vivo. Propagation in culture may alter expression of some secreted proteins compared to a native tissue context. In addition, the keratinocyte secretome is dynamic, and we know that the landscape of secreted proteins will change in states of inflammation or disease. Additionally, as part of our filtering strategy, we intersected our experimentally detected proteins with the list of all secreted proteins reported in the Human Protein Atlas project [70]. While this strategy removes false-positive intracellular protein contaminants released from low-level cell lysis, it may exclude true-positive secreted proteins. The Human Protein Atlas designates secreted proteins based on presence of signal peptides; proteins secreted by nonclassical pathways, such as IL-1 [71, 72], would be excluded using these criteria.

As a class, secreted proteins are more readily detected in body fluids and tissue samples, making them valuable as potential biomarkers and molecular signatures of disease [73, 74]. Secreted proteins and extracellular membrane regions are critical to active medical therapies, and represent the target for nearly 70% of current US Food and Drug Administration-approved drugs [70]. However, for skin conditions, laboratory biomarkers are less developed than for other organs and tissues, leaving an opportunity for further development in dermatology and skin-related health conditions.

In summary, we experimentally defined the repertoire of the human epidermal keratinocyte secretome. Further, we developed a colony formation-based CRISPR screen and identified SFRP1 as a key inhibitor of keratinocyte progenitor proliferation. Mechanistically, SFRP1 functions in epidermal stemness regulation through suppressing LIF expression in addition to Wnt signaling. The results and screening approach presented in this work can provide a strategy for identifying and functionally studying secreted proteins that can contribute to better diagnosis and treatment of skin disease.

## Materials and methods

### Primary human epidermal keratinocytes

Primary human neonatal keratinocytes were isolated from discarded foreskin tissues harvested with informed consent under an approved UC Irvine Institutional Review Board protocol. Keratinocytes were cultured in a 50:50 mixture of Keratinocyte-SFM and 154 media (Thermo Fisher Scientific, Waltham, MA, USA, cat#10724-011 and cat#M-154-500) with manufacturer-recommended supplements and 1x Antibiotic-Antimycotic. 293 T cells and 3T3 cells were cultured in DMEM medium supplemented with 10% fetal bovine serum and 1x Antibiotic-Antimycotic. All cells were cultured at 37 °C at 5% CO_2_.

### Collection of conditioned media and protein identification

Primary neonatal human epidermal keratinocytes, pooled from three unrelated donors, were seeded in 150 mm tissue culture dishes. For the undifferentiated progenitor state, keratinocytes were seeded at 5 × 10^6^ cells per dish and maintained in subconfluent conditions for 48 h. For differentiation of keratinocytes, 3 ×10^7^ keratinocytes were seeded onto a 150 mm dish and visualized 12 h later to confirm full confluence. The media was supplemented to 1.2 mM calcium with calcium chloride. The confluent cells were maintained for 8 days, replacing calcium-supplemented media every two days. Conditioned media from both undifferentiated and differentiated conditions were harvested 48 h after media change. Media was centrifuged at 500 × g for 10 min to pellet cell debris. The supernatant was collected and concentrated through a low protein-binding polyethersulfone column with 3 K cutoff (MacroSep, Pall Life Sciences, Port Washington, NY, USA). The concentrated conditioned media were loaded onto a well of a 4–12% gradient, 1.0 mm Bis-Tris NuPAGE gel and resolved by electrophoresis. Equivalent total supernatant protein mass was loaded for each lane. The gel was stained with Coomassie Blue (Thermo Fisher). Each lane of protein lysate was divided into 9 gel slices of equal area and provided for proteomics analysis. Proteins were resolved by micro capillary reverse phase HPLC directly coupled to the nano-electrospray ionization source of an LTQ-Orbitrap mass spectrometer (µLC-MS/MS). The µLC-MS/MS and the spectra analysis were performed by the Harvard Mass Spectrometry and Proteomics Resource Laboratory (Cambridge, MA, USA) to generate the list of proteins. A false discovery rate of 0.1 was applied based on a reverse database strategy, and background contaminants were excluded based on the facility’s repository of common background proteins in human proteomics experiments.

### Secretome GO term analysis

To assess for Gene Ontology enrichment, the secretome gene list was analyzed by the NIH Database for Annotation, Visualization and Integrated Discovery (DAVID) tool. The background list for analysis was comprised of 15,129 genes expressed in control keratinocyte RNA-seq results, defined as those genes meeting a minimum expression threshold of >1 count based on a geometric average of all samples.

### Cas9-expressing keratinocyte cell line

To generate a Cas9-expressing keratinocyte cell line, the Cas9-Flag-P2A-puromycin cassette was removed from the pLentiCRISPRv2 plasmid and cloned into the pLEX MCS vector digested with BamHI and XhoI. Next, the puromycin resistance cassette was swapped for a blasticidin resistance cassette, generating the vector pLEX-Cas9-Blast. Lentivirus was generated with pLEX-Cas9-Blast and infected into the clone 103 keratinocyte cell line [75]. After selection in blasticidin, selected keratinocytes were plated at low dilution to expand individual keratinocyte clones. After expansion, cell colonies were isolated and further propagated. Protein lysates of each clone were evaluated by FLAG immunoblot (anti-FLAG, Cell Signaling, Danvers, MA, USA, cat#14793, 1:1000) to identify clones with the highest levels of Cas9 expression. A high-expressing clone (103-Cas9 B2 clone 11, herein referred in this manuscript as Cas9-keratinocyte) was used for the CRISPR screen. The cellular growth and differentiation behavior of Cas9-keratinocyte was tested to verify its ability to form clones in a clonogenic assay, its capacity for differentiation when plated to confluence and elevated calcium, and its comparable staining to primary keratinocytes for b-galactosidase, as a proxy marker for senescence.

### CRISPR secretome sgRNA library design

Cas9 guide sequences targeting the 118 secreted protein candidates, 12 non targeting controls, and core essential genes (EIF3A, RPL11, RPLP2, U2AF1) were extracted from the Brunello human sgRNA library [27]. Four independent sgRNAs for each candidate and control gene were used in the library. For all sgRNAs a “G” was prepended to the sgRNA sequence to facilitate transcription by the Pol III promoter. Synthesized oligonucleotides were ordered as a pool (IDT).

### CRISPR secretome sgRNA library construction

The sgRNA oligo pool was resuspended in IDTE buffer pH 8.0 at a concentration of 400 ng/µl. To convert the oligos to double-stranded DNA and generate homologous overhangs for InFusion cloning, a PCR was performed with primers of CRISPR sgRNA-fwd and CRISPR sgRNA-rev (Supplementary Table 1), with the protocol of [98 °C × 10 s, 58 °C × 10 s, 72 °C × 10 s] 6 cycles. The PCR product was purified by column purification and cloned into linearized pSICO-(F + E) vector restriction digested with Esp3I and Smil at a molar ratio of 10:1 insert:vector. The total copy number of cloned insert for the InFusion reaction was determined to exceed 1000x coverage of the CRISPR secretome library to maintain library complexity. Transformed E. coli competent cells were incubated with SOC medium with shaking for 1 h at 37 °C and transferred to LB medium to incubate overnight with shaking. The plasmid library pool was harvested by maxiprep, and next-generation sequencing of the library was performed to confirm library diversity. Sequencing confirmed presence of 499 out of 500 library sgRNAs.

### CRISPR secretome lentiviral library production

The CRISPR secretome lentiviral library was produced in 293T cells with polyethyeneimine (PEI) transfection of the CRISPR secretome sgRNA library plasmids with packaging plasmids pUC-MDG and pCMV-Δ8.91. Lentiviral-containing supernatant was harvested 72 h after transfection, passed through a low protein-binding filter, and concentrated 30 times (v/v) using Lenti-X concentrator. Functional viral titer was established by performing titrations of virus on Cas9-keratinocytes and assessing infection efficiency by viable cell survival after puromycin treatment. A multiplicity of infection of 0.16 was used for the CRISPR screen.

### Colony formation assay

For quantification of stem cell potential, keratinocytes were seeded at low density on a bed of feeder fibroblasts and cultivated to form stem cell colonies over 12 days. To prepare feeders, 3T3 fibroblasts were first treated with mitomycin C (15 µg/ml) for 2 h at 37 °C to block cell division. The feeder layer was prepared by plating 4.6 × 10^6^ mitomycin C-treated fibroblasts onto 10-cm dishes and incubated overnight. The next day, 10,000 secretome CRISPR library-infected keratinocytes were seeded onto each dish in KGM (Keratinocyte Growth Medium). KGM medium is composed of 10% fetal bovine serum, adenine at 2.7 µg/ml, cholera toxin at 2.8 ng/ml, hydrocortisone at 40 ng/ml, insulin at 4.94 µg/ml, recombinant human EGF at 10 ng/ml, 0.1% v/v T/T3 mix, and 1X antibiotic-antimycotic. T/T3 mix was made by combining 1 volume of T (5 mg/ml) with 99 volume of T3 (136 ng/ml).

Keratinocyte colonies were cultured for 12 days with KGM media replaced every 3 days. At the endpoint, 3T3 cells were removed by washing with DPBS. For the CRISPR screen, colonies from twenty-five 10-cm dishes were pooled and collected, and genomic DNA harvested.

### Analysis of CRISPR screen results

To assess sgRNA abundance at each timepoint, genomic DNA was amplified using two step PCR to append Illumina compatible sequences. The first PCR reaction (PCR1) was performed using 4.3 µg (1200x library coverage) of genomic DNA from each timepoint using a mixture of forward primers (PCR1-fwd01+fwd02+fwd03+fwd04) and a reverse primer (PCR1-rev). The forward primers contain a mixture of random (N) nucleotides added to generate a sequence stagger and improve complexity and clustering during early steps of Illumina sequencing. Primer list is shown in Supplementary Table 1. PCR1 was performed with the protocol: 98 °C 2:00, [98 °C 10 s, 55 °C 15 s, 68 °C 20 s] 10 cycles.

The second PCR step (PCR2) was performed using the purified PCR1 product as a template. Each condition was amplified with the same PCR2-fwd primer but had unique reverse primer barcodes to permit multiplexing (D709-D712). PCR2 was performed with the protocol: 98 °C 2:00, [98 °C 10 s, 55 °C 15 s, 68 °C 20 s] 8 cycles.

PCR2 amplification products were gel-purified and quantified with NEBNext Library Quantification Kit for Illumina, followed by the NGS sequencing (NovaSeq 6000). NGS sequencing results were analyzed by CRISPRCloud2 (https://crispr.nrihub.org). The FASTA file of the 500 guide RNAs in the CRISPR secretome library and FASTQ files of samples in the screen (day0_replicate1, day0_replicate2, day12_replicate1, day12_replicate2) were the input of the analysis, and Survival and Dropout screen type was selected.

### RNA interference-mediated gene knockdown

For short hairpin-targeted gene knockdown of SFRP1, shRNAs were cloned into the linearized pLKO.1 vector digested with AgeI and EcoRI. The hairpin sequences are listed in Supplementary Table 2. Lentivirus was produced in 293 T cells with polyethyeneimine (PEI) as described above. For infection, 5 × 10^5^ primary keratinocytes were infected with shControl or shSFRP1 in 50:50 media containing 3 μg/ml polybrene. After overnight incubation, cells were grown in keratinocyte media supplemented with 1 μg/ml puromycin for 72 h.

### sgRNA-mediated gene knockout

For sgRNA-targeted gene knockout of SFRP1 or LIF via CRISPR-Cas9, sgRNAs were cloned into linearized pSICO-(F + E) vector restriction digested with Esp3I and Smil. The sgRNA sequences are listed in Supplementary Table 2.

For lentivirus co-infection in primary keratinocytes, lentivirus was combined prior to infection: 50% shControl + 50% shControl, 50% shControl+ 50% shSFRP1, 50% shControl + 50% shLIF or shCTNNB1, and 50% shSFRP1 + 50% shLIF or CTNNB1. The total amount of lentivirus and cell number were the same as that used for single target infections.

### Colony formation assay and crystal violet staining

6 × 10^5^ mitomycin C-treated 3T3 were seeded onto one well of 6-well plate and incubated overnight. The next day, 1200 keratinocytes were seeded onto each well. Clones were propagated for 12 days with media changed every 3 days. At the endpoint, fibroblasts were dislodged by washing with DPBS. Colonies were fixed in methanol: acetone (1:1) for 5 min, air-dried for 5 min, and stained with 0.02% crystal violet for 3 min. Cells were air-dried and imaged. Colony area and number counting were performed using ImageJ software, with 1000 square pixels set as a minimum threshold to exclude non-colony staining.

### Supernatant, RNA, and protein collection

Cells were seeded onto 6-well (for day 0) or 24-well (for days 1-4 of differentiation) plates at a density of 2.5 × 10^5^ cells/well. Media were changed the following day with 50:50 media (for day 0) or 50:50 media supplemented with 1.2 mM calcium (for days 1–4). At each timepoint the supernatant and cells were harvested, and cell debris depleted from supernatants by centrifugation at 1000 × *g* for 10 min.

RNA was harvested for qPCR analysis using primers shown in Supplementary Table 3.

Protein lysates were harvested with RIPA buffer and quantitated with BCA Protein Assay kit. Antibodies used in western blot were: anti-beta-tubulin (DSHB, Iowa City, IA, USA, cat#E7, 1:1000), anti-SFRP1(Cell signaling, cat#3534, 1:1000), anti-LIF (Proteintech, Rosemont, IL, USA, cat# 26757-1-AP, 1:300), anti-β-Catenin (Cell signaling, cat#4270, 1:1000), anti-FOXM1 (Cell signaling, cat#20459, 1:1000), IRDye 680RD Goat anti-rabbit **(**LI-COR, Lincoln, NE, USA, cat#926-68071, 1:10,000), IRDye 800CW Donkey anti-mouse (LI-COR, cat#926-32212, 1:10,000).

Supernatant was used in ELISA for SFRP1 detection (Abcam, Fremont, CA, USA, cat#Ab277082). Supernatant concentrated through Nanosep with 3 K Omega Centrifugal Filters (Pall, cat#0D003C33) was used in western blot for SFRP1 detection and in ELISA for LIF detection (Abcam, cat#ab242228).

### Organotypic culture

Air-dried devitalized human dermis was mounted onto 1.7-cm × 1.7-cm supports, and 5 × 10^5^ keratinocytes were seeded onto the basement membrane. Tissue was grown in KGM media at an air-liquid interface for 7 days, with media changed every 2 days. For shControl and shSFRP1 infected primary keratinocytes, half of the tissue was collected in Buffer RLT Plus, shredded with Mini-BeadBeater (BIOSPEC, Bartlesville, OK, USA), and followed by total RNA isolation. Half of the tissue was embedded in O.C.T. Compound, sectioned on a cryostat at 7-μm thickness, visualized with hematoxylin and eosin staining or immunofluorescence staining.

### Recombinant human protein treatment

For rhSFRP1 treatment, recombinant Human sFRP-1 (bio-techne R&D systems, Minneapolis, MN, USA, cat#5396-SF) was added to KGM media at a concentration of 1 ng/ml. For rhLIF treatment, recombinant Human LIF (bio-techne R&D systems, cat#7734-LF) was added to KGM media of colony formation assay at a concentration of 20 ng/ml. Recombinant protein was refreshed with each media change.

### Hematoxylin and eosin staining

Sections were fixed with 4% formaldehyde for 10 min, followed by incubation with 0.2% Triton-X 100 in PBS for 5 min. Hematoxylin incubation was applied for 12 min, followed by tap water, 1% acid alcohol, tap water, 0.2% ammonia, tap water, and final wash with 95% ethanol. Eosin was applied for 30 s, followed by two washes with 100% ethanol, and two washes of xylene. Dried slides were mounted with permount mounting medium and visualized with an EVOS M5000 Imaging System.

Quantification of the basal curvature was performed by Kappa-Curvature Analysis in Fiji [76]. Open B-Spline was chosen as the input type. A total of thirty H&E sections for shControl and shSFRP1 were measured, from three biological replicates, with ten images samples from each replicate.

### Immunofluorescence staining

Organotypic culture tissue sections were mounted onto polysine slides. Sections were fixed for 10 min in 4% formaldehyde in PBS for KRT10, methanol for FLG, or acetone for LOR and Ki67. Slides were blocked in PBS-Tween (0.1%) with 5% normal goat serum for 1 h. Sections were incubated with primary antibodies in PBS-Tween (0.1%) with 2% serum overnight at 4 °C. Then incubated with secondary antibodies in PBS-Tween (0.1%) with 2% serum for 45 min at RT. Slides were washed with PBS-Tween (0.1%) three times, incubated with Hoechst stain (Cell signaling, cat#4082) for 2 min, washed with PBST and Milli-Q water, then mounted with Prolong Gold antifade reagent (Invitrogen, cat#P36934) and coverslips. Antibodies were: anti-Cytokeratin 10 (Abcam, cat#76318, 1:1000), anti-filaggrin (Santa Cruz, Santa Cruz, CA, USA, cat#sc-66192, 1:200), anti-loricrin (BioLegend, San Diego, CA, USA, cat#90501, 1:200), anti-Ki67 (Invitrogen, cat#MA5-14520, 1:00). Alexa Fluor 555 goat anti-rabbit and goat anti-mouse (Thermo Fisher Scientific, cat#A21428 and cat#A21422, 1:500). Slides were visualized with EVOS M5000 Imaging System. Ki67 positive cells were counted by ImageJ software.

Immunofluorescence staining was also performed on cells (for β-galactosidase, Cell Signaling, cat# 27198 and cleaved caspase-3, Cell Signaling, cat#9664) or colonies (for Ki67) seeded on chamber slides.

### RNA-sequencing

Primary keratinocytes of two independent biological replicates were infected with shControl or shSFRP1 and selected for 72 h with puromycin. Total RNA was harvested using RNeasy Plus Mini Kit and sequenced on an Illumina NovaSeq 6000 with 150-nucleotide paired-end reads to a depth of >17 million reads per sample, achieving a minimal mean quality score above 35. RNA-seq analysis was performed in Partek Flow. The input of the fastq files (day0_replicate1, day0_replicate2, day2_replicate1, day2_replicate2) was trimmed from both ends to a minimal quality score of 30. Trimmed reads were aligned to the human reference genome (hg38) using STAR. Gene expression was quantified to the hg38 Ensembl release 105 annotation, and differential analysis was performed by Gene Specific Analysis, applying a low count filter of 1 and default normalization methods of CPM (counts per million). Differentially expressed genes (DEGs) were filtered based on a threshold of *p* ≤ 0.05 and |log_2_(fold change)| > 1. For the background list for Gene Ontology analysis, genes that had at least one count in at least one sample, as well as a geometric average of gene counts of all samples > 1.0 were included.

### Assay for transposase-accessible chromatin with sequencing

Primary keratinocytes from two independent biological replicates were infected with shControl or shSFRP1 and selected for 72 h with puromycin. 5 × 10^4^ cells were pelleted and lysed using 50 µl lysis buffer. The mixture was washed with dilution buffer and centrifuged at 500 × *g* for 10 min at 4 °C. The supernatant was aspirated and the cell pellet was resuspended in 50 µl of transposition mixture and incubated at 37 °C for 30 min in a thermomixer. Transposition fragments were isolated using the Zymo DNA Clean and Concentrator-5 kit.

Eluted DNA was amplified with 2.5 µl of 25 µM primer Ad1.noMX, 2.5 µl of 25 µM primer Ad2.Index with barcode, and 25 µl of 2x NEBNext master mix, with the protocol: 72 °C for 5 min, 98 °C for 30 s, 98 °C for 10 s, 63 °C for 30 s, 72 °C for 1 min, with steps 3–5 repeated 4 times. The primers are in Supplementary Table 4. Quantitative PCR was performed to determine the optimal number of PCR cycles using an aliquot of the pre-amplified mixture [77, 78]. The remaining pre-amplified mixture was further amplified using the optimal number of cycles and purified using the Zymo DNA Clean and Concenrtrator-5 kit.

Library quantification was performed using the NEBNext Library Quant for Illumina and sequenced on NovaSeq S4 platform with paired-end reads to a depth of >80 million reads per sample with a minimal mean quality score >34.

### Analysis of ATAC-sequencing

ATAC-seq analysis was performed in Partek Flow. The input of the FSTQ files (shControl_replicate1, shControl_replicate2, shSFRP1_replicate1, shSFRP1_replicate2) were aligned to Homo sapiens(human)-hg38, index Ensembl Transcripts release 105 with BWA-MEM. Aligned reads were filtered for duplicates, and MACS was used to call peaks. The peaks were quantified to regions with strict paired-end compatibility, with minimum region size of 50 and minimum 50% of base overlap. The region counts were normalized then annotated with hg38, annotation model Ensembl Transcripts release 105. Differential analysis was performed using Gene Specific Analysis with lowest average coverage filter of 1, and differential regions were filtered by a threshold of *p* ≤ 0.05 and |fold change| > 1.5 (shSFRP1 vs. shControl). Gene set enrichment was performed on differential regions with hg38, gene set database 2022_02_01, with the same background gene list used for RNA-sequencing analysis.

### Single-cell RNA sequencing analysis

Single-cell transcriptomics datasets of human neonatal foreskin epidermis (GSE147482) [79] were downloaded from the NCBI GEO database (https://www.ncbi.nlm.nih.gov/geo/). The raw count matrix was generated using the CellRanger Pipeline (version 2.1.0, 10x Genomics), aligned to the human reference genome (GRCh38). The Seurat R package (v4.3.0) was used to combine all cell libraries into a merged Seurat object. Genes detected in <3 Cells were removed. Low-quality cells were further filtered based on sample-specific QC metrics, with thresholds applied to individual samples: - Replicates 1, 2, 4, and 5: Cells with >500 and <5000 detected genes per cell, <30,000 UMI counts per cell, and <15% mitochondrial gene expression was retained; Replicate 3: Cells with >500 and <6,000 detected genes per cell, <50,000 UMI counts per cell, and <20% mitochondrial gene expression was included in the analysis.

Single-cell data from each sample was processed for doublet detection. Raw counts were normalized using the NormalizeData() function with a scale factor of 10,000, and variable features were identified using FindVariableFeatures() with 2000 genes. Principal component analysis (PCA) was performed, and the first 30 principal components were used for dimensionality reduction via uniform manifold approximation and projection (UMAP). Clustering was conducted using the FindNeighbors() and FindClusters() functions with 30 PCA components and a resolution parameter of 0.5. The doublets were predicted with DoubletFinder (v2.0) [80]. No discrete doublet-enriched cluster was identified and few doublets were observed in the dataset.

For visualization, the five samples were integrated using the FindIntegrationAnchors() and IntegrateData() functions with default parameters. UMAP visualization and clustering were performed on 30 principal components with a resolution parameter of 0.5. The expression levels of canonical marker genes were used to annotate biological cell types within each cluster of the total population, including *KRT5, KRT14*, and *COL17A1* (basal keratinocytes, BAS); *MKI67* (proliferating basal keratinocytes, BAS-P1/P2); *KRT1* and *KRT10* (spinous keratinocytes, SPN); *SPINK5* (granular keratinocytes, GRN); *PMEL* (melanocytes, MEL); *SOSTDC1* (appendage-related cells, APD); *PECAM1* (endothelial cells, EC); and *CD207* (Langerhans cells, LC).

To assess the effects of cell cycle heterogeneity on basal cell states, cell cycle phase annotation was performed using Seurat’s CellCycleScoring() function with cell cycle gene sets provided by Seurat.

### Statistical analysis

Immunoblot bands, immunofluorescent staining, colony formation area and number were quantitated with ImageJ software (version 1.54i). Quantification of epidermal organoid tissues features was performed by ImageJ software (for Ki67-positive cell count, epidermal area) and Fiji software (version 2.14.0/1.54 f, for basal curvature). The details of the quantification are indicated in the methods.

Data are presented as the mean ± standard deviation (SD) or standard error (SEM), or median ± interquartile range. For experiments reporting SEM, replicates are primary epidermal keratinocytes derived from distinct biological donors. For two group comparisons, statistical analysis for significance was determined using Student *t*-test with a threshold of *p* ≤ 0.05 considered to be significant. For comparisons involving three or more groups, statistical analysis for significance was determined using one-way ANOVA with a Tukey’s HSD post hoc test with a threshold of *p* ≤ 0.05 considered to be significant. GraphPad Prism 10 (GraphPad Software) was used to execute statistical comparisons, with significant *p* values presented. The details of statistical analysis are indicated in the figure legends.

## Supplementary information

Supplemental Figure Legends

Figure S1A-B

Figure S1C

Figure S2

Figure S3

Figure S4

Figure S5

Supplemental Table 1

Supplemental Table 2

Supplemental Table 3

Supplemental Table 4

Supplemental Table 5

Supplemental Table 6

Original Western Blots

## Data Availability

RNA sequencing and ATAC sequencing data are deposited at NIH Gene Expression Omnibus with the accession numbers GSE269627 and GSE269626, respectively.

## References

[1] Lopez-Pajares V, Yan K, Zarnegar BJ, Jameson KL, Khavari PA. Genetic pathways in disorders of epidermal differentiation. Trends Genet. 2013;29:31–40. 23141808 10.1016/j.tig.2012.10.005 PMC5477429

[2] Anderton H, Alqudah S. Cell death in skin function, inflammation, and disease. Biochem J. 2022;479:1621–51. 35929827 10.1042/BCJ20210606 PMC9444075

[3] Orsmond A, Bereza-Malcolm L, Lynch T, March L, Xue M. Skin barrier dysregulation in psoriasis. Int J Mol Sci. 2021;22:10841. 34639182 10.3390/ijms221910841 PMC8509518

[4] Hsu Y-C, Fuchs E. Building and maintaining the skin. Cold Spring Harb Perspect Biol. 2022;14:a040840. 34607830 10.1101/cshperspect.a040840 PMC8977401

[5] Townsend EC, Kalan LR. The dynamic balance of the skin microbiome across the lifespan. Biochem Soc Trans. 2023;51:71–86. 36606709 10.1042/BST20220216 PMC9988004

[6] Khan AQ, Agha MV, Sheikhan KSAM, Younis SM, Tamimi MA, Alam M, et al. Targeting deregulated oxidative stress in skin inflammatory diseases: an update on clinical importance. Biomedicine Pharmacother. 2022;154:113601. 10.1016/j.biopha.2022.113601 36049315

[7] Pfisterer K, Shaw LE, Symmank D, Weninger W. The extracellular matrix in skin inflammation and infection. Front Cell Dev Biol. 2021;9. 10.3389/fcell.2021.682414. 10.3389/fcell.2021.682414 PMC8290172 34295891

[8] Wu W, Krijgsveld J. Secretome analysis: reading cellular sign language to understand intercellular communication. Mol Cell Proteom. 2024;23. 10.1016/j.mcpro.2023.100692. 10.1016/j.mcpro.2023.100692 PMC10793180 38081362

[9] Noske K. Secreted immunoregulatory proteins in the skin. J Dermatol Sci. 2018;89:3–10. 29111181 10.1016/j.jdermsci.2017.10.008

[10] Lim X, Tan SH, Koh WLC, Chau RMW, Yan KS, Kuo CJ, et al. Interfollicular epidermal stem cells self-renew via autocrine Wnt signaling. Science. 2013;342:1226–30. 24311688 10.1126/science.1239730 PMC4081860

[11] Liarte S, Bernabé-García Á, Nicolás FJ. Role of TGF-β in skin chronic wounds: a keratinocyte perspective. Cells. 2020;9:306. 32012802 10.3390/cells9020306 PMC7072438

[12] El-Serafi AT, El-Serafi I, Steinvall I, Sjöberg F, Elmasry M. A systematic review of keratinocyte secretions: a regenerative perspective. Int J Mol Sci. 2022;23:7934. 35887279 10.3390/ijms23147934 PMC9323141

[13] Igawa S, Choi JE, Wang Z, Chang Y-L, Wu C-C, Werbel T, et al. Human keratinocytes use sphingosine 1-phosphate and its receptors to communicate staphylococcus aureus invasion and activate host defense. J Investig Dermatol. 2019;139:1743–1752.e5. 30807768 10.1016/j.jid.2019.02.010 PMC7682680

[14] Hu Y, Guo J, Yin L, Tu J, Yin Z. Tacrolimus inhibits TNF-α/IL-17A-produced pro-inflammatory effect on human keratinocytes by regulating IκBζ. Inflammation. 2020;43:692–700. 31838663 10.1007/s10753-019-01151-6

[15] Daneshmandi L, Shah S, Jafari T, Bhattacharjee M, Momah D, Saveh-Shemshaki N, et al. Emergence of the stem cell secretome in regenerative engineering. Trends Biotechnol. 2020;38:1373–84. 32622558 10.1016/j.tibtech.2020.04.013 PMC7666064

[16] Md Fadilah NI, Mohd Abdul Kader Jailani MS, Badrul Hisham MAI, Sunthar Raj N, Shamsuddin SA, Ng MH, et al. Cell secretomes for wound healing and tissue regeneration: next generation acellular-based tissue-engineered products. J Tissue Eng. 2022;13:20417314221114273. 35923177 10.1177/20417314221114273 PMC9340325

[17] Katz AB, Taichman LB. A partial catalog of proteins secreted by epidermal keratinocytes in culture. J Investig Dermatol. 1999;112:818–21. 10233778 10.1046/j.1523-1747.1999.00572.x

[18] Simons BD, Clevers H. Strategies for homeostatic stem cell self-renewal in adult tissues. Cell. 2011;145:851–62. 21663791 10.1016/j.cell.2011.05.033

[19] Flora P, Ezhkova E. Regulatory mechanisms governing epidermal stem cell function during development and homeostasis. Development. 2020;147:dev194100. 33191273 10.1242/dev.194100 PMC7687856

[20] Wagner RN, Piñón Hofbauer J, Wally V, Kofler B, Schmuth M, De Rosa L, et al. Epigenetic and metabolic regulation of epidermal homeostasis. Exp Dermatol. 2021;30:1009–22. 33600038 10.1111/exd.14305 PMC8359218

[21] Blanpain C, Fuchs E. Epidermal homeostasis: a balancing act of stem cells in the skin. Nat Rev Mol Cell Biol. 2009;10:207–17. 19209183 10.1038/nrm2636 PMC2760218

[22] Barrandon Y, Green H. Three clonal types of keratinocyte with different capacities for multiplication. Proc Natl Acad Sci USA. 1987;84:2302–6. 2436229 10.1073/pnas.84.8.2302 PMC304638

[23] Enzo E, Secone Seconetti A, Forcato M, Tenedini E, Polito MP, Sala I, et al. Single-keratinocyte transcriptomic analyses identify different clonal types and proliferative potential mediated by FOXM1 in human epidermal stem cells. Nat Commun. 2021;12:2505. 33947848 10.1038/s41467-021-22779-9 PMC8097075

[24] Handly LN, Pilko A, Wollman R. Paracrine communication maximizes cellular response fidelity in wound signaling. eLife. 2015;4:e09652. 26448485 10.7554/eLife.09652 PMC4686426

[25] Li L, Wang BH, Wang S, Moalim-Nour L, Mohib K, Lohnes D, et al. Individual cell movement, asymmetric colony expansion, Rho-associated kinase, and E-cadherin impact the clonogenicity of human embryonic stem cells. Biophys J. 2010;98:2442–51. 20513387 10.1016/j.bpj.2010.02.029 PMC2877320

[26] Francis K, Palsson BO. Effective intercellular communication distances are determined by the relative time constants for cyto/chemokine secretion and diffusion. Proc Natl Acad Sci USA. 1997;94:12258–62. 9356436 10.1073/pnas.94.23.12258 PMC24899

[27] Doench JG, Fusi N, Sullender M, Hegde M, Vaimberg EW, Donovan KF, et al. Optimized sgRNA design to maximize activity and minimize off-target effects of CRISPR-Cas9. Nat Biotechnol. 2016;34:184–91. 26780180 10.1038/nbt.3437 PMC4744125

[28] Hart T, Brown KR, Sircoulomb F, Rottapel R, Moffat J. Measuring error rates in genomic perturbation screens: gold standards for human functional genomics. Molecular Syst Biol. 2014;10:733. 10.15252/msb.20145216 PMC4299491 24987113

[29] Inoue M, Chang L, Hwang J, Chiang S-H, Saltiel AR. The exocyst complex is required for targeting of Glut4 to the plasma membrane by insulin. Nature. 2003;422:629–33. 12687004 10.1038/nature01533

[30] Esposito G, Vitagliano L, Costanzo P, Borrelli L, Barone R, Pavone L, et al. Human aldolase A natural mutants: relationship between flexibility of the C-terminal region and enzyme function. Biochem J. 2004;380:51–56. 14766013 10.1042/BJ20031941 PMC1224144

[31] Wang L, Wu D, Robinson CV, Wu H, Fu T-M. Structures of a complete human V-ATPase reveal mechanisms of its assembly. Mol Cell. 2020;80:501–511.e3. 33065002 10.1016/j.molcel.2020.09.029 PMC7655608

[32] Zhang M, Liu L, Lin X, Wang Y, Li Y, Guo Q, et al. A translocation pathway for vesicle-mediated unconventional protein secretion. Cell. 2020;181:637–652.e15. 32272059 10.1016/j.cell.2020.03.031

[33] Li Y, Zhang C, Zhang H, Feng W, Wang Q, Fan R. Severe phenotypes of B3GAT3-related disorder caused by two heterozygous variants: a case report and literature review. BMC Med Genomics. 2022;15:27. 35151321 10.1186/s12920-022-01160-9 PMC8841085

[34] Filippou PS, Karagiannis GS, Constantinidou A. Midkine (MDK) growth factor: a key player in cancer progression and a promising therapeutic target. Oncogene. 2020;39:2040–54. 31801970 10.1038/s41388-019-1124-8

[35] Liang C-J, Wang Z-W, Chang Y-W, Lee K-C, Lin W-H, Lee J-L. SFRPs are biphasic modulators of Wnt-signaling-elicited cancer stem cell properties beyond extracellular control. Cell Rep. 2019;28:1511–1525.e5. 31390565 10.1016/j.celrep.2019.07.023

[36] Sun J-Z, Zhang J-H, Li J-B, Yuan F, Tong L-Q, Wang X-Y, et al. MXRA5 is a novel immune-related biomarker that predicts poor prognosis in glioma. Dis Markers. 2021;2021:6680883. 34211612 10.1155/2021/6680883 PMC8211501

[37] Xavier CP, Melikova M, Chuman Y, Üren A, Baljinnyam B, Rubin JS. Secreted Frizzled-related protein potentiation versus inhibition of Wnt3a/β-catenin signaling. Cell Signal. 2014;26:94–101. 24080158 10.1016/j.cellsig.2013.09.016 PMC3953133

[38] Sunkara RR, Mehta D, Sarate RM, Waghmare SK. BMP-AKT-GSK3β signaling restores hair follicle stem cells decrease associated with loss of Sfrp1. Stem Cells. 2022;40:802–17. 35689817 10.1093/stmcls/sxac041

[39] Choi YS, Zhang Y, Xu M, Yang Y, Ito M, Peng T, et al. Distinct functions for Wnt/β-catenin in hair follicle stem cell proliferation and survival and interfollicular epidermal homeostasis. Cell Stem Cell. 2013;13:720–33. 24315444 10.1016/j.stem.2013.10.003 PMC3900235

[40] Tripurani SK, Wang Y, Fan Y-X, Rahimi M, Wong L, Lee M-H, et al. Suppression of Wnt/β-catenin signaling by EGF receptor is required for hair follicle development. Mol Biol Cell. 2018;29:2784–99. 30188763 10.1091/mbc.E18-08-0488 PMC6249831

[41] Liu J, Zhu H, Wang H, Li J, Han F, Liu Y, et al. Methylation of secreted frizzled-related protein 1 (SFRP1) promoter downregulates Wnt/β-catenin activity in keloids. J Mol Hist. 2018;49:185–93. 10.1007/s10735-018-9758-3 29455276

[42] Liang J, Liu L, Tang H, Ma Q, Sang Y, Kang X. UVB-induced SFRP1 methylation potentiates skin damage by promoting cell apoptosis and DNA damage. Exp Dermatol. 2022;31:1443–53. 35657114 10.1111/exd.14621

[43] Sunkara RR, Sarate RM, Setia P, Shah S, Gupta S, Chaturvedi P, et al. SFRP1 in Skin tumor initiation and cancer stem cell regulation with potential implications in epithelial cancers. Stem Cell Rep. 2020;14:271–84. 10.1016/j.stemcr.2019.12.006 PMC7013199 31928951

[44] Bencomo T, Lee CS. Gene expression landscape of cutaneous squamous cell carcinoma progression. Br J Dermatol. 2024;191:760–774. 10.1093/bjd/ljae249 38867481

[45] Yao Y, Richman L, Morehouse C, de los Reyes M, Higgs BW, Boutrin A, et al. Type I interferon: potential therapeutic target for psoriasis?. PLoS ONE. 2008;3:e2737. 18648529 10.1371/journal.pone.0002737 PMC2481274

[46] Correa da Rosa J, Malajian D, Shemer A, Rozenblit M, Dhingra N, Czarnowicki T, et al. Patients with atopic dermatitis have attenuated and distinct contact hypersensitivity responses to common allergens in skin. J Allergy Clin Immunol. 2015;135:712–20. 25583101 10.1016/j.jaci.2014.11.017

[47] Han F, Konkalmatt P, Mokashi C, Kumar M, Zhang Y, Ko A, et al. Dopamine D2 receptor modulates Wnt expression and control of cell proliferation. Sci Rep. 2019;9:16861. 31727925 10.1038/s41598-019-52528-4 PMC6856370

[48] The Gene Ontology Consortium, Aleksander SA, Balhoff J, Carbon S, Cherry JM, Drabkin HJ, et al. The gene ontology knowledgebase in 2023. Genetics. 2023;224:iyad031. 36866529 10.1093/genetics/iyad031 PMC10158837

[49] Ashburner M, Ball CA, Blake JA, Botstein D, Butler H, Cherry JM, et al. Gene ontology: tool for the unification of biology. Nat Genet. 2000;25:25–29. 10802651 10.1038/75556 PMC3037419

[50] Levine AJ, Puzio-Kuter AM, Chan CS, Hainaut P. The role of the p53 protein in stem-cell biology and epigenetic regulation. Cold Spring Harb Perspect Med. 2016;6:a026153. 27352800 10.1101/cshperspect.a026153 PMC5008064

[51] Ge Y, Wang J, Zhang H, Li J, Ye M, Jin X. Fate of hematopoietic stem cells determined by Notch1 signaling (Review). Exp Ther Med. 2022;23:170. 35069851 10.3892/etm.2021.11093 PMC8764575

[52] Jing J, Wu Z, Wang J, Luo G, Lin H, Fan Y, et al. Hedgehog signaling in tissue homeostasis, cancers, and targeted therapies. Sig Transduct Target Ther. 2023;8:1–33. 10.1038/s41392-023-01559-5 PMC10439210 37596267

[53] Hu J, Katayama H, Nakagawa H, Ono S, Imai T, Shimizu N. Leukemia inhibitory factor induces epidermal hyperplasia in patients with amyotrophic lateral sclerosis. J Investig Dermatol. 2000;115:486–92. 10951288 10.1046/j.1523-1747.2000.00047.x

[54] McKenzie RC, Szepietowski J. Cutaneous leukemia inhibitory factor and its potential role in the development of skin tumors. Dermatol Surg. 2004;30:279–90. 14871222 10.1111/j.1524-4725.2004.30087.x

[55] Bai R, Guo Y, Liu W, Song Y, Yu Z, Ma X. The roles of WNT signaling pathways in skin development and mechanical-stretch-induced skin regeneration. Biomolecules. 2023;13:1702. 38136575 10.3390/biom13121702 PMC10741662

[56] Liu T, Jia P, Ma H, Reed SA, Luo X, Larman HB, et al. Construction and screening of a lentiviral secretome library. Cell Chem Biol. 2017;24:767–771.e3. 28602759 10.1016/j.chembiol.2017.05.017

[57] Ding M, Tegel H, Sivertsson Å, Hober S, Snijder A, Ormö M, et al. Secretome-based screening in target discovery. SLAS Discov. 2020;25:535–51. 32425085 10.1177/2472555220917113 PMC7309359

[58] Lin H, Lee E, Hestir K, Leo C, Huang M, Bosch E, et al. Discovery of a cytokine and its receptor by functional screening of the extracellular proteome. Science. 2008;320:807–11. 18467591 10.1126/science.1154370

[59] Jennbacken K, Wågberg F, Karlsson U, Eriksson J, Magnusson L, Chimienti M, et al. Phenotypic screen with the human secretome identifies FGF16 as inducing proliferation of ipsc-derived cardiac progenitor cells. Int J Mol Sci. 2019;20:6037. 31801200 10.3390/ijms20236037 PMC6928864

[60] van Asten SD, Raaben M, Nota B, Spaapen RM. Secretome screening reveals fibroblast growth factors as novel inhibitors of viral replication. J Virol. 2018;92:e00260–18. 29899088 10.1128/JVI.00260-18 PMC6069191

[61] Gonzalez R, Jennings LL, Knuth M, Orth AP, Klock HE, Ou W, et al. Screening the mammalian extracellular proteome for regulators of embryonic human stem cell pluripotency. Proc Natl Acad Sci USA. 2010;107:3552–7. 20133595 10.1073/pnas.0914019107 PMC2840467

[62] Harbinski F, Craig VJ, Sanghavi S, Jeffery D, Liu L, Sheppard KA, et al. Rescue screens with secreted proteins reveal compensatory potential of receptor tyrosine kinases in driving cancer growth. Cancer Discov. 2012;2:948–59. 22874768 10.1158/2159-8290.CD-12-0237

[63] Popp T, Steinritz D, Breit A, Deppe J, Egea V, Schmidt A, et al. Wnt5a/β-catenin signaling drives calcium-induced differentiation of human primary keratinocytes. J Investig Dermatol. 2014;134:2183–91. 24658506 10.1038/jid.2014.149

[64] Mendoza-Reinoso V, Beverdam A. Epidermal YAP activity drives canonical WNT16/β-catenin signaling to promote keratinocyte proliferation in vitro and in the murine skin. Stem Cell Res. 2018;29:15–23. 29562208 10.1016/j.scr.2018.03.005

[65] Fukunaga-Kalabis M, Hristova DM, Wang JX, Li L, Heppt MV, Wei Z, et al. UV-induced Wnt7a in the human skin microenvironment specifies the fate of neural crest -like cells via suppression of Notch. J Investig Dermatol. 2015;135:1521–32. 25705850 10.1038/jid.2015.59 PMC4430391

[66] Tsai SL, Baselga-Garriga C, Melton DA. Midkine is a dual regulator of wound epidermis development and inflammation during the initiation of limb regeneration. eLife. 2020;9:e50765. 31934849 10.7554/eLife.50765 PMC6959999

[67] Monma F, Hozumi Y, Ikematsu S, Kawaguchi M, Kadomatsu K, Suzuki T. Expression of midkine in normal human skin, dermatitis and neoplasms: association with differentiation of keratinocytes. J Dermatol. 2013;40:980–6. 24304120 10.1111/1346-8138.12333

[68] Inazumi T, Tajima S, Nishikawa T, Kadomatsu K, Muramatsu H, Muramatsu T. Expression of the retinoid-inducible polypeptide, midkine, in human epidermal keratinocytes. Arch Dermatol Res. 1997;289:471–5. 9266025 10.1007/s004030050223

[69] Peng S, Zhu X, Zhao M, Zhang Y, Wang A, Chen M, et al. Identification of matrix-remodeling associated 5 as a possible molecular oncotarget of pancreatic cancer. Cell Death Dis. 2023;14:1–14. 36828810 10.1038/s41419-023-05684-5 PMC9958022

[70] Uhlén M, Fagerberg L, Hallström BM, Lindskog C, Oksvold P, Mardinoglu A, et al. Tissue-based map of the human proteome. Science. 2015;347:1260419. 25613900 10.1126/science.1260419

[71] Daniels MJD, Brough D. Unconventional pathways of secretion contribute to inflammation. Int J Mol Sci. 2017;18:102. 28067797 10.3390/ijms18010102 PMC5297736

[72] Cohen MJ, Chirico WJ, Lipke PN. Through the back door: unconventional protein secretion. Cell Surf. 2020;6:100045. 33225116 10.1016/j.tcsw.2020.100045 PMC7666356

[73] Bang H, Kim JE, Lee HS, Park SM, Park D-J, Lee EJ. Integrated bioinformatic analysis of gene expression profiling data to identify combinatorial biomarkers in inflammatory skin disease. Sci Rep. 2022;12:5889. 35393522 10.1038/s41598-022-09840-3 PMC8989986

[74] Stastna M, Van Eyk JE. Secreted proteins as a fundamental source for biomarker discovery. Proteomics. 2012;12:722–35. 22247067 10.1002/pmic.201100346 PMC3517109

[75] Cai P, Otten ABC, Cheng B, Ishii MA, Zhang W, Huang B, et al. A genome-wide long noncoding RNA CRISPRi screen identifies *PRANCR* as a novel regulator of epidermal homeostasis. Genome Res. 2020;30:22–34. 31804951 10.1101/gr.251561.119 PMC6961571

[76] Schindelin J, Arganda-Carreras I, Frise E, Kaynig V, Longair M, Pietzsch T, et al. Fiji: an open-source platform for biological-image analysis. Nat Methods. 2012;9:676–82. 22743772 10.1038/nmeth.2019 PMC3855844

[77] Buenrostro JD, Giresi PG, Zaba LC, Chang HY, Greenleaf WJ. Transposition of native chromatin for fast and sensitive epigenomic profiling of open chromatin, DNA-binding proteins and nucleosome position. Nat Methods. 2013;10:1213–8. 24097267 10.1038/nmeth.2688 PMC3959825

[78] Buenrostro J, Wu B, Chang H, Greenleaf W. ATAC-seq: a method for assaying chromatin accessibility genome-wide. Curr Protoc Mol Biol. 2015;109:21.29.1–21.29.9. 25559105 10.1002/0471142727.mb2129s109 PMC4374986

[79] Wang S, Drummond ML, Guerrero-Juarez CF, Tarapore E, MacLean AL, Stabell AR, et al. Single cell transcriptomics of human epidermis identifies basal stem cell transition states. Nat Commun. 2020;11:4239. 32843640 10.1038/s41467-020-18075-7 PMC7447770

[80] McGinnis CS, Murrow LM, Gartner ZJ. DoubletFinder: doublet detection in single-cell RNA sequencing data using artificial nearest neighbors. Cell Syst. 2019;8:329–337.e4. 30954475 10.1016/j.cels.2019.03.003 PMC6853612

