# Supplementary material for: Comprehensive secretome profiling and CRISPR screen identifies SFRP1 as a key inhibitor of epidermal progenitor proliferation
Source: Cell Death Dis. 2025 May 3;16(1):360. doi: 10.1038/s41419-025-07691-0 (PMC12049499; doi:10.1038/s41419-025-07691-0)
Supplement: Supplementary file 1 — Supplemental Figure Legends [file 41419_2025_7691_MOESM1_ESM.docx]

**Supplementary Figure 1. The human epidermal keratinocyte secretome**.

1. Quantitative RT-PCR to measure markers of progenitor and differentiated keratinocytes to evaluate status of keratinocytes used for conditioned media/mass spectrometry analysis.
2. Identified proteins labeled as contaminants in the mass spectrometry data and their respective spectral counts from the output data.
3. List of 406 proteins that comprise the human epidermal keratinocyte secretome, as identified by mass spectrometry of conditioned media. Proteins are color coded to indicate their detection in progenitor/undifferentiated (purple), differentiated (teal), or both states (yellow). Raw spectral counts in progenitor (P) and differentiated (D) conditions are shown.

**Supplementary Figure 2. Characteristics and quality control of secretome CRISPR screen.**

1. Immunofluorescence of β-galactosidase in primary keratinocytes and Cas9-expressing keratinocytes. Scale bar, 150 µm.
2. Quantification of β-galactosidase positive cells in (A). Each datapoint represents the average integrated density of β-galactosidase per cell from a sampled image. Mean value of primary keratinocyte was set to 1 for comparation. Data are mean with range. (n=5, evaluated with two-tailed unpaired student’s t-test).
3. Quantitative RT-PCR of differentiation-associated genes (KRT1, KRT10, FLG, LOR), and progenitor-associated genes (BNC1, CCNB1, CCND1, CYR61) in primary keratinocytes across time course of *in vitro* differentiation. Data are mean ± SD. (n=4, two-way ANOVA with a Tukey’s HSD post hoc test).
4. Quantitative RT-PCR of differentiation-associated and progenitor-associated genes in Cas9 expressing keratinocytes across time course of *in vitro* differentiation. Data are mean ± SD. (n=4, two-way ANOVA with a Tukey’s HSD post hoc test).
5. Colony morphology of the CRISPR screen screening endpoint day 12.
6. Coverage of secretome CRISPR library. NGS sequencing confirmed a 99.8% coverage of the 500 sgRNAs and 100% coverage of the 123 targets of the library in all samples (day 0 replicate1 and 2, day 12 replicate1 and 2).
7. Violin plot of day 0 sgRNA counts. Average CPM (counts per million) of the sgRNA replicates for each target at day0 is displayed. Lines represent median and quartiles.
8. Scatter plot of day 0 (left) and day 12 (right) sgRNA abundance. Normalized log_2_CPM (counts per million) of all sgRNA in two replicates is displayed. Each dot represents the abundance of a single sgRNA in two replicate samples.

**Supplementary Figure 3. Characteristics of SFRP1 knockdown human keratinocytes and keratinocyte colonies.**

1. Immunoblot of SFRP1 protein depletion in Cas9-keratinocytes. Four independent SFRP1 sgRNAs in CRISPR screen, and two independent control sgRNAs are displayed. Relative intensity of SFRP1 blot is denoted. sgControl_1 was set to 1 for comparation.
2. Crystal violet staining of Cas9-keratinocyte colonies generated following treatment with sgControl_1, sgSFRP1_2 and sgSFRP1_3.
3. Quantification of colony formation area in (B). Mean of sgControl was set to 1 for comparison. Data are means ± SD. (n=6, one-way ANOVA with a Tukey’s HSD post hoc test).
4. Quantification of colony formation number in (B). Mean of sgControl was set to 1 for comparison. Data are means ± SD. (n=6, one-way ANOVA with a Tukey’s HSD post hoc test).
5. Quantitative RT-PCR of SFRP1 mRNA depletion in primary keratinocytes. SFRP1_201, isoform SFRP1_201; SFRP1, total isoforms. Data are mean ± SEM. (n=4, multiple paired student’s t-test).
6. Immunofluorescence for cleaved caspase-3 in control and SFRP1 knockdown keratinocyte colonies. Caspase-3 signal was only detected in the surrounding mitomycin-C treated 3T3 cells but not within the keratinocyte colonies of both shControl and shSFRP1. Scale bar, 300 µm.
7. Quantitative RT-PCR of SFRP1 mRNA isoforms in primary keratinocytes differentiated *in vitro* (day 0 to day 4). SFRP1 can be generated in two isoforms. SFRP1_201, isoform SFRP1_201; SFRP1, all isoforms. Data are mean ± SEM. (n=4, one-way ANOVA with a Tukey’s HSD post hoc test, p-values <0.05 are specified.).
8. ELISA of SFRP1 in primary keratinocytes across time course of *in vitro* differentiation. Data are mean ± SEM. Day 0 expression was set to 1 for comparison. (n=3, one-way ANOVA with a Tukey’s HSD post hoc test, p-values <0.05 are specified).
9. Immunoblot of SFRP1 in keratinocyte whole cell lysate and supernatant across a time course of *in vitro* differentiation. Cell lysate immunoblot signal was normalized to beta tubulin. Supernatant immunoblot signal was normalized to total protein. Day 0 protein expression was set to 1 for comparison.

**Supplementary Figure 4. GO analysis of RNA-seq, TSS plot and GO analysis of ATAC-seq for SFRP1 KD.**

1. Top enriched biological process (BP) and molecular function (MF) GO terms (ranked by p-value) of DEGs on both day 0 and day 2.
2. List of genes involved in day 0 Wnt GO (p-value ≤0.05), with fold change (shSFRP1 vs. shControl) and p-value in RNA-seq denoted.
3. List of genes involved in day 2 Wnt GO (p-value ≤0.05), with fold change (shSFRP1 vs. shControl) and p-value in RNA-seq denoted. Only top genes (ranked by fold change) are shown.
4. List of genes involved in day 0 Stem GO, with fold change (shSFRP1 vs. shControl) and p-value denoted. Genes also involved in Wnt GO are colored in red.
5. List of genes involved in day 2 Stem GO, with fold change (shSFRP1 vs. shControl) and p-value denoted. Only top genes (ranked by fold change) are shown. Genes also involved in Wnt GO are colored in red.
6. TSS (Transcription start site) plot in ATAC-seq. The read coverage around the transcription start sites within the detected peaks were displayed. Top, TSS profile plot; bottom, TSS heatmap. The plot encompasses 1 kb around peak centers.
7. Top enriched biological process (BP) and molecular function (MF) GO (ranked by p-value) of differential peak-associated genes in ATAC-seq.

**Supplementary Figure 5. Characteristics of LIF in normal and SFRP1 knockdown keratinocytes.**

1. Counts of transcripts in RNA-seq. Normalized CPM (counts per million) of LIF transcripts in RNA-seq samples in two replicates at day 0 and day 2. The counts of LIF transcripts are compared with SFRP1, and two differentiation markers KRT10 and FLG.
2. Quantitative RT-PCR of LIF mRNA in primary keratinocytes at day 0 to day 4. LIF_201, isoform LIF_201; LIF, all isoforms. Data are mean ± SEM. (n=4, one-way ANOVA with a Tukey’s honestly significant differences [HSD] post hoc test).
3. Immunoblot of LIF protein in control or SFRP1-depleted primary keratinocytes at day 0 to day 4. Relative intensity of SFRP1 blot is denoted. Day 4 was set to 1 for comparison.
4. ELISA of LIF protein in primary keratinocytes at day 0 to day 4. Day 0 was set to 1 for comparison. Data are mean ± SEM. (n=3, one-way ANOVA with a Tukey’s honestly significant differences [HSD] post hoc test).
5. Quantification of colonies in Figure 6F. The average of shControl replicates was set to 1. Data are means ± SD (n=6, one-way ANOVA with a Tukey’s honestly significant differences [HSD] post hoc test).
6. Quantification of colonies in Figure 6J. The average of shControl replicates was set to 1. Data are means ± SD (n=6, one-way ANOVA with a Tukey’s honestly significant differences [HSD] post hoc test).
