# Supplementary figures and images for: Comprehensive secretome profiling and CRISPR screen identifies SFRP1 as a key inhibitor of epidermal progenitor proliferation
Source: Cell Death Dis. 2025 May 3;16(1):360. doi: 10.1038/s41419-025-07691-0 (PMC12049499; doi:10.1038/s41419-025-07691-0)

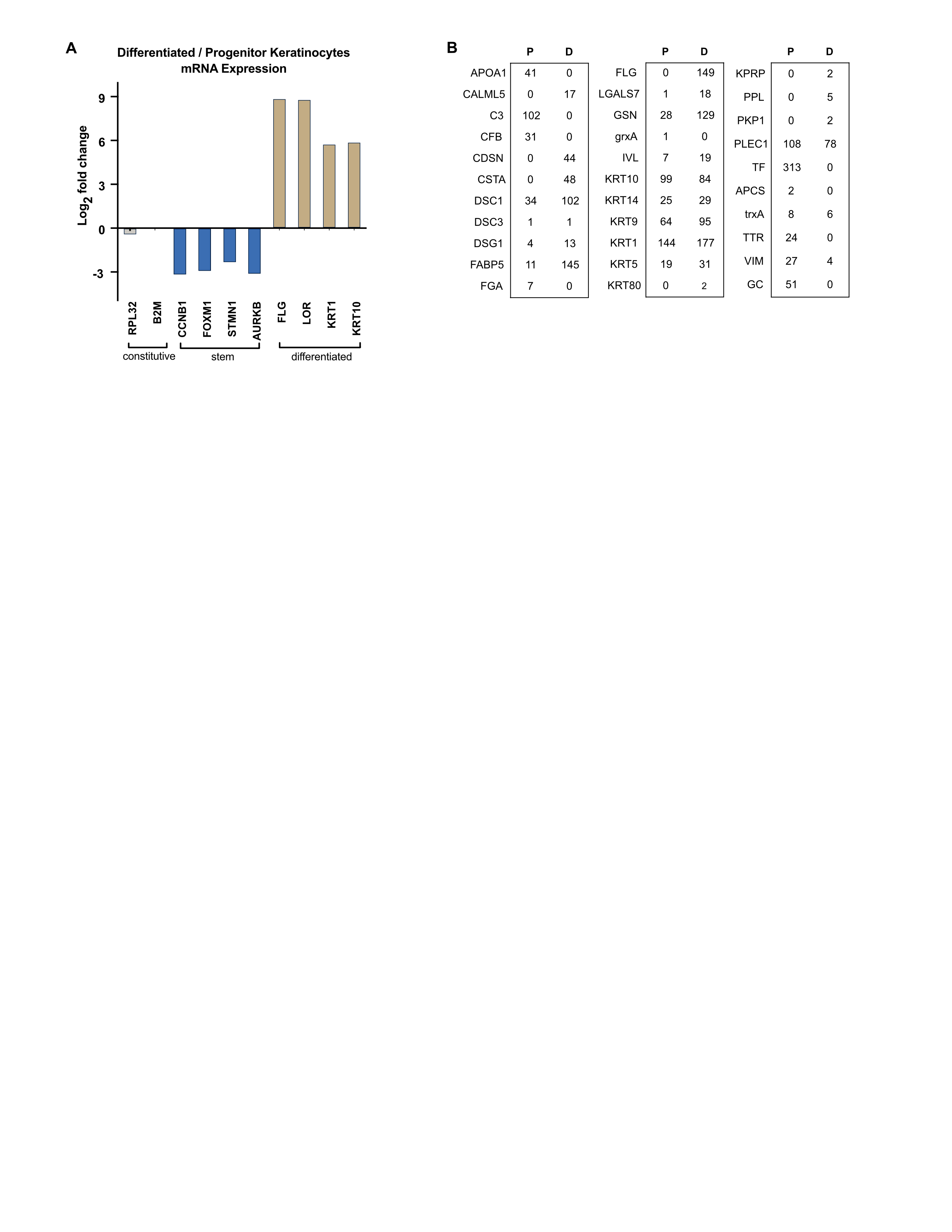

Supplement: Supplementary file 2 — Figure S1A-B [file 41419_2025_7691_MOESM2_ESM.tif]

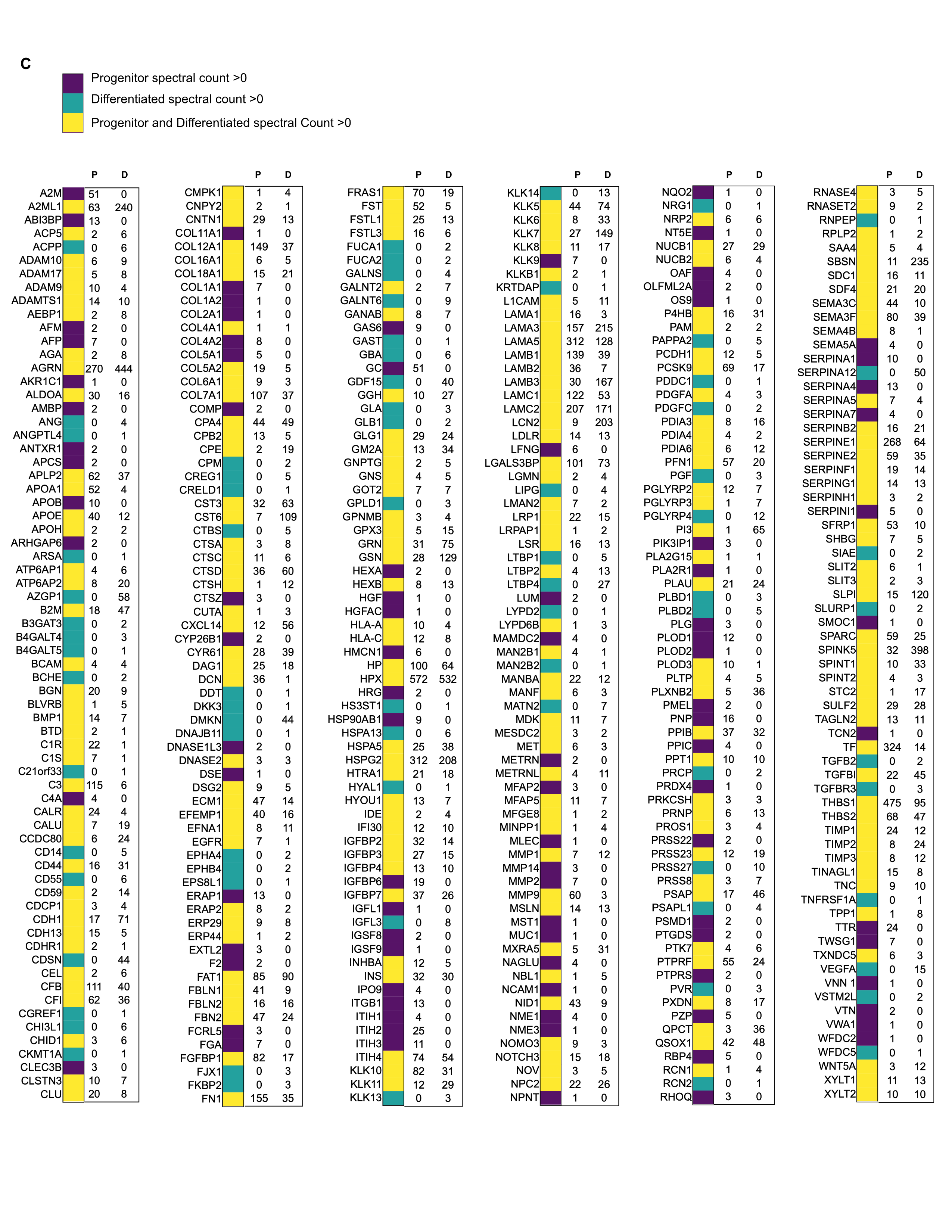

Supplement: Supplementary file 3 — Figure S1C [file 41419_2025_7691_MOESM3_ESM.tif]

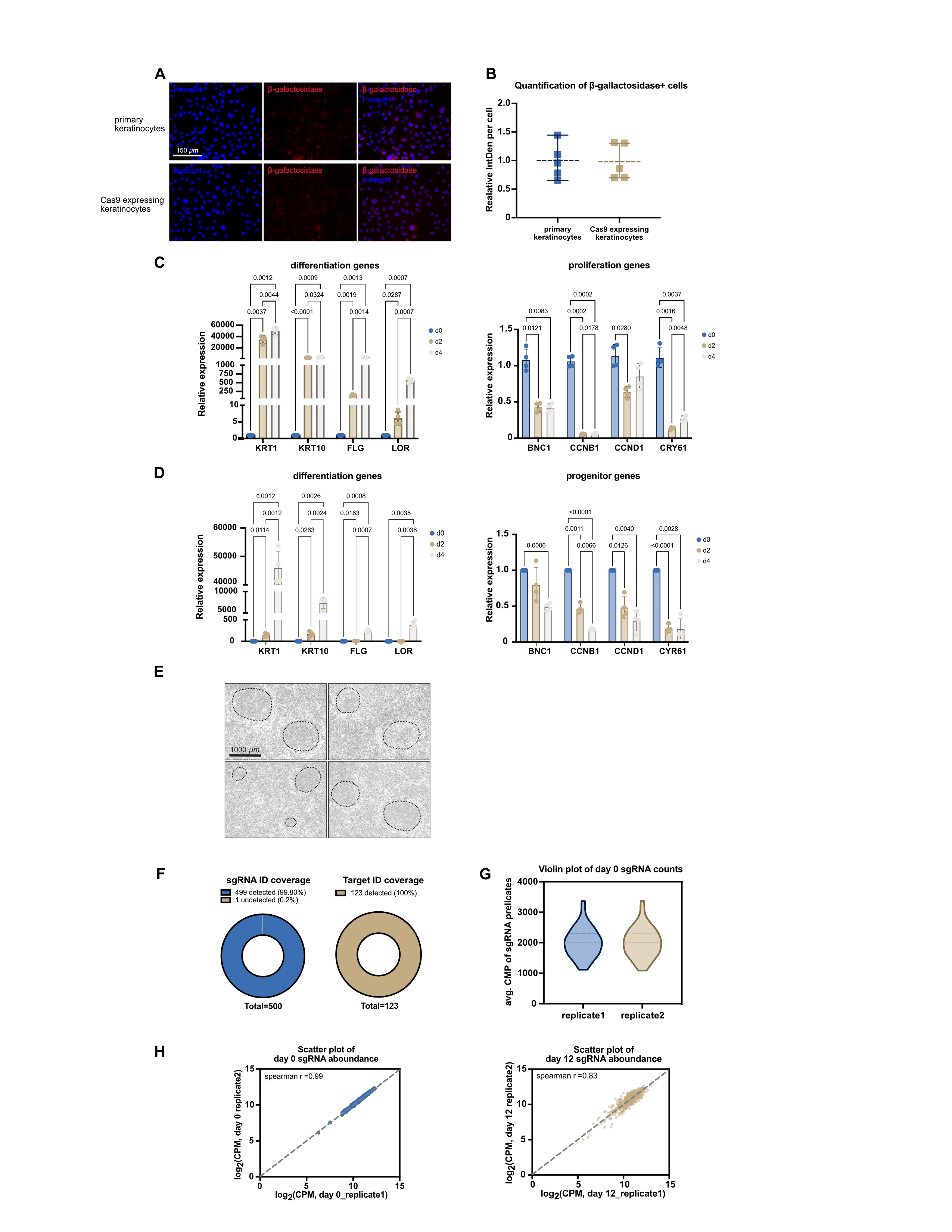

Supplement: Supplementary file 4 — Figure S2 [file 41419_2025_7691_MOESM4_ESM.tif]

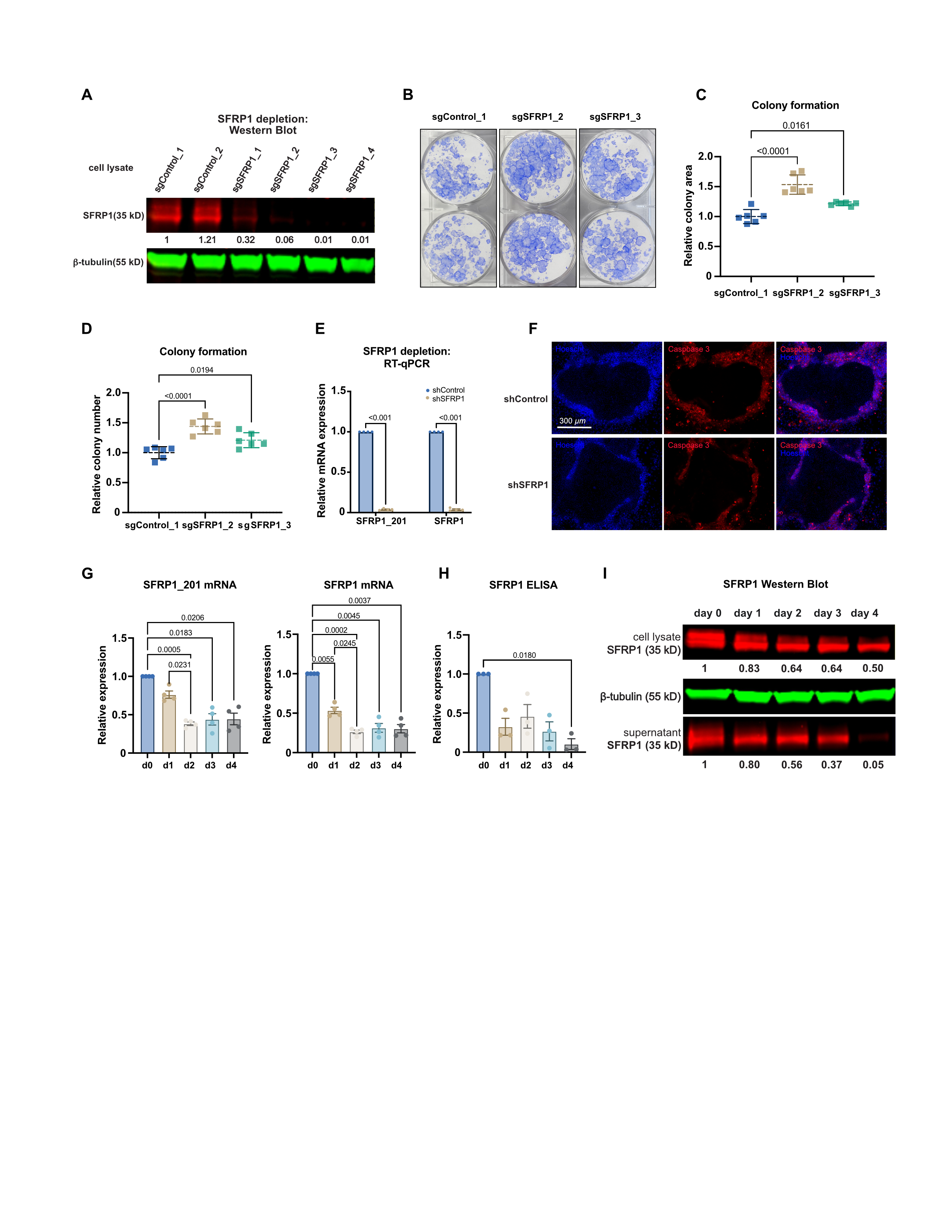

Supplement: Supplementary file 5 — Figure S3 [file 41419_2025_7691_MOESM5_ESM.tif]

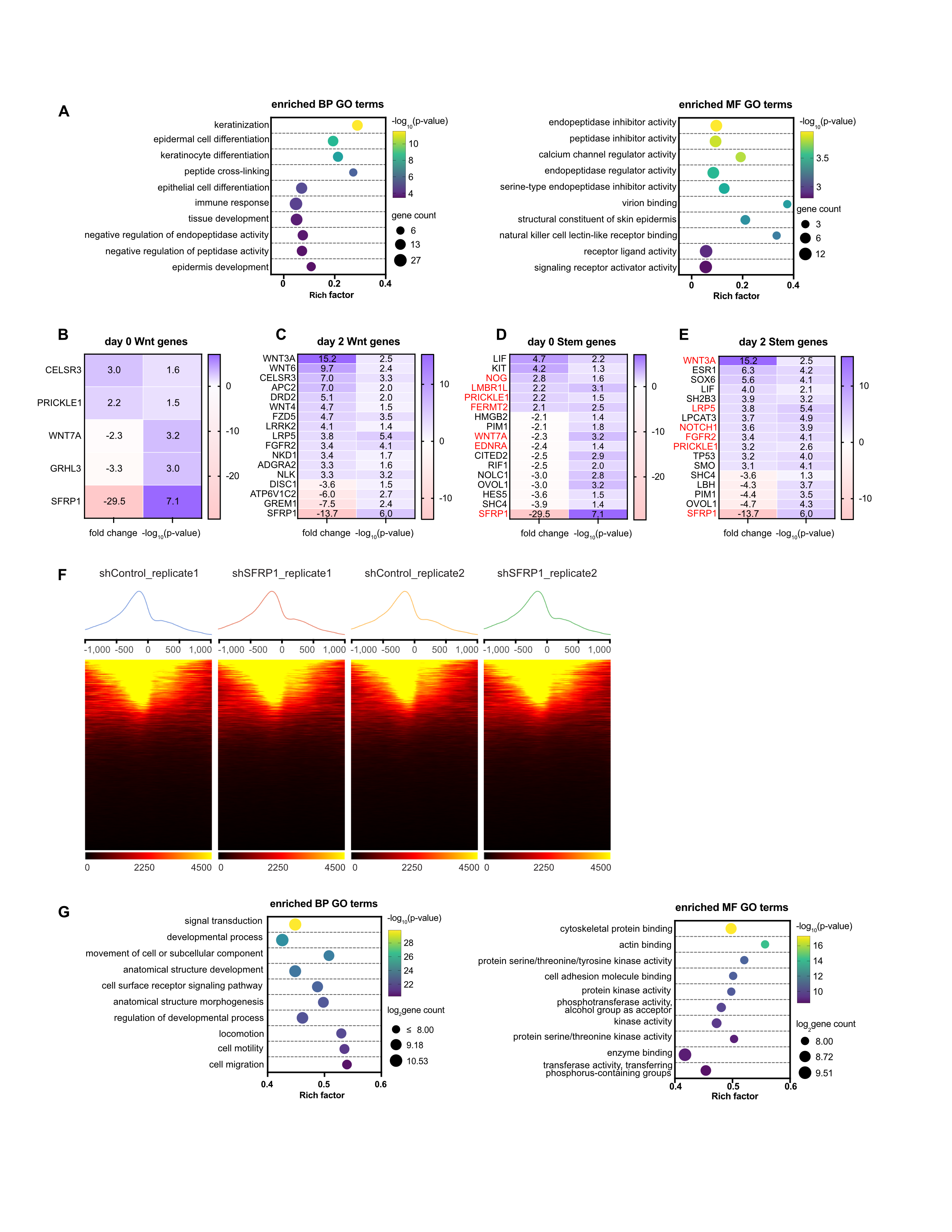

Supplement: Supplementary file 6 — Figure S4 [file 41419_2025_7691_MOESM6_ESM.tif]

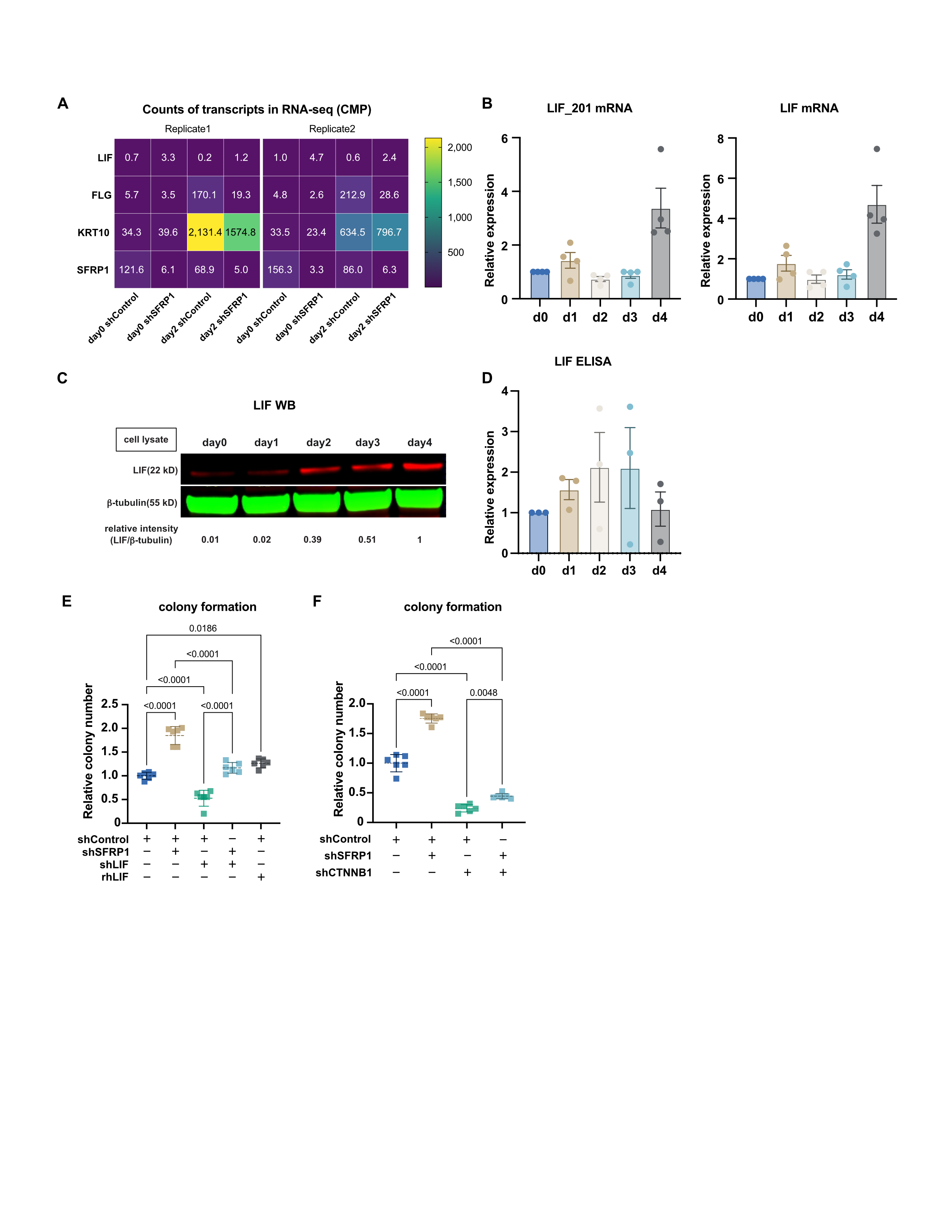

Supplement: Supplementary file 7 — Figure S5 [file 41419_2025_7691_MOESM7_ESM.tif]

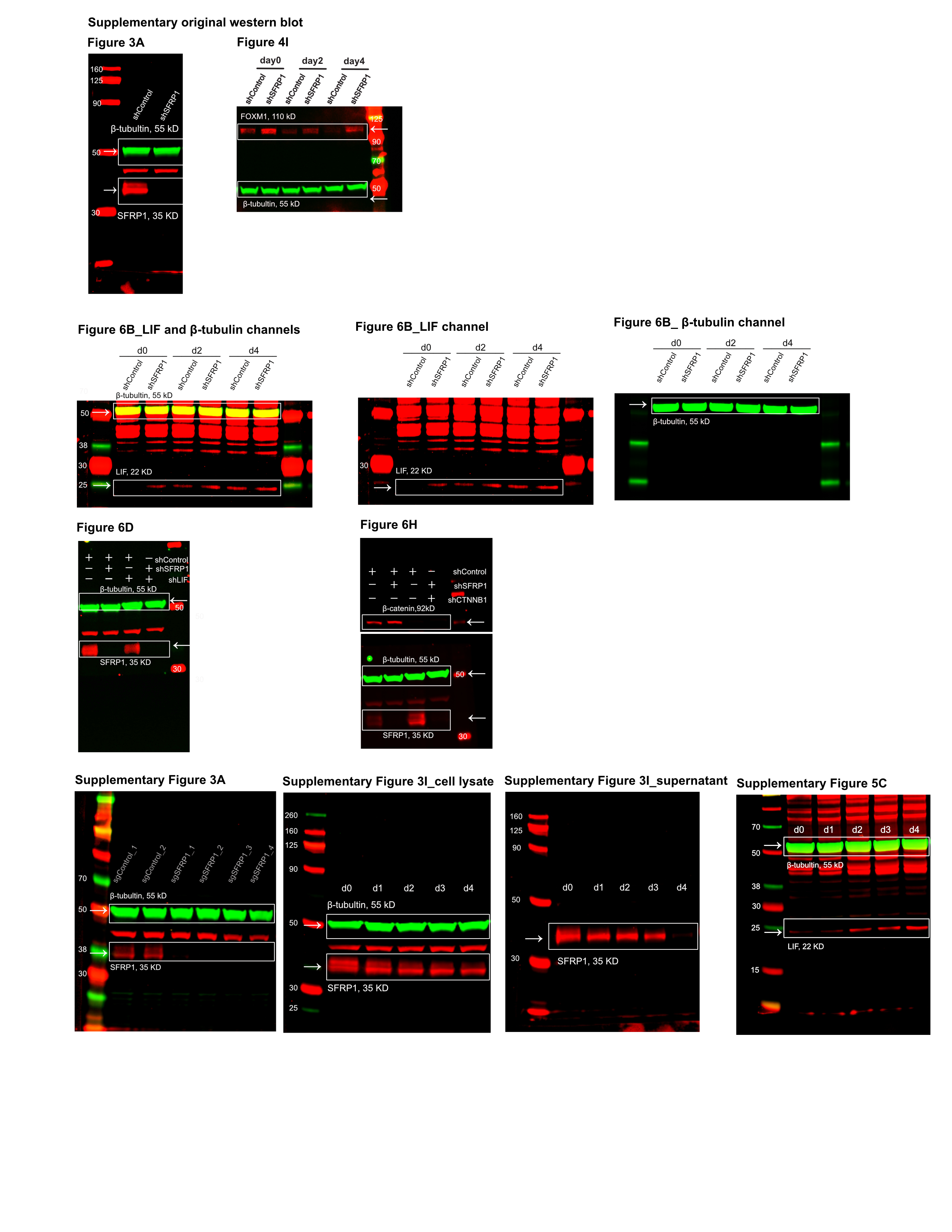

Supplement: Supplementary file 14 — Original Western Blots [file 41419_2025_7691_MOESM14_ESM.tif]
