# Supplementary material for: Comprehensive secretome profiling and CRISPR screen identifies SFRP1 as a key inhibitor of epidermal progenitor proliferation
Source: Cell Death Dis. 2025 May 3;16(1):360. doi: 10.1038/s41419-025-07691-0 (PMC12049499; doi:10.1038/s41419-025-07691-0)
Supplement: Supplementary file 8 — Supplemental Table 1 [file 41419_2025_7691_MOESM8_ESM.docx]

**Supplementary Table 1. CRISPR screen primer list.**

| **Name** | **Primer sequences (5’ > 3’)** |
| --- | --- |
| CRISPR sgRNA-fwd | GTGGAAAGGACGAAACACC |
| CRISPR sgRNA-rev | TTCCAGCATAGCTCTTAAAC |
| PCR1-fwd01 | ACACGACGCTCTTCCGATCTTGTGGAAAGGACGAAACACC |
| PCR1-fwd02 | ACACGACGCTCTTCCGATCTNNTGTGGAAAGGACGAAACACC |
| PCR1-fwd03 | ACACGACGCTCTTCCGATCTNNNNTGTGGAAAGGACGAAACACC |
| PCR1-fwd04 | ACACGACGCTCTTCCGATCTNNNNNNTGTGGAAAGGACGAAACACC |
| PCR1-rev | GTGACTGGAGTTCAGACGTGTGCTCTTCCGATCTGCATGGCGGTAATACGGTTATCC |
| PCR2-fwd no index | AATGATACGGCGACCACCGAGATCTACACTCTTTCCCTACACGACGCTCTTCCGATCT |
| PCR2_rev_D709 | CAAGCAGAAGACGGCATACGAGATCATAGCCGGTGACTGGAGTTCAGACGTG |
| PCR2_rev_D710 | CAAGCAGAAGACGGCATACGAGATTTCGCGGAGTGACTGGAGTTCAGACGTG |
| PCR2_rev_D711 | CAAGCAGAAGACGGCATACGAGATGCGCGAGAGTGACTGGAGTTCAGACGTG |
| PCR2_rev_D712 | CAAGCAGAAGACGGCATACGAGATCTATCGCTGTGACTGGAGTTCAGACGTG |
