# Supplementary material for: Comprehensive secretome profiling and CRISPR screen identifies SFRP1 as a key inhibitor of epidermal progenitor proliferation
Source: Cell Death Dis. 2025 May 3;16(1):360. doi: 10.1038/s41419-025-07691-0 (PMC12049499; doi:10.1038/s41419-025-07691-0)
Supplement: Supplementary file 9 — Supplemental Table 2 [file 41419_2025_7691_MOESM9_ESM.docx]

**Supplementary Table 2. shRNA and sgRNA oligo list.**

| **Name** | **Oligo sequences (5’ > 3’)** |
| --- | --- |
| Control shRNA | CCTAAGGTTAAGTCGCCCTCG |
| SFRP1 shRNA | GAGAGTTATCCTGATAAATTA |
| LIF shRNA | AGTGCCAATGCCCTCTTTATT |
| CTNNB1 shRNA | CCTAGCCTTGCTTGTTAAATT |
| Control sgRNA_1 | AGCGTCTAACGTGGCGCTC |
| Control sgRNA_2 | CTGACGCGACGGTAGCACA |
| SFRP1 sgRNA_1 | AGTGTGACAAGTTCCCCGAG |
| SFRP1 sgRNA_2 | CGACTACGTGAGCTTCCAGT |
| SFRP1 sgRNA_3 | CTCAACAAGAACTGCCACGC |
| SFRP1 sgRNA_4 | TCAGTGCGTGGACATCCCCG |
