# Supplementary material for: Comprehensive secretome profiling and CRISPR screen identifies SFRP1 as a key inhibitor of epidermal progenitor proliferation
Source: Cell Death Dis. 2025 May 3;16(1):360. doi: 10.1038/s41419-025-07691-0 (PMC12049499; doi:10.1038/s41419-025-07691-0)
Supplement: Supplementary file 10 — Supplemental Table 3 [file 41419_2025_7691_MOESM10_ESM.docx]

**Supplementary Table 3. qPCR primer list.**

| **Name** | **Primer sequences (5’ > 3’)** |
| --- | --- |
| SFRP1_201_fwd | TGGCCCGAGATGCTTAAGTG |
| SFRP1_201_rev | CTCGTTGTCACAGGGAGGAC |
| SFRP1_fwd | ATCTCTGTGCCAGCGAGTTT |
| SFRP1_rev | GTCAGCCCCATTCTTCAGGT |
| LIF_201_fwd | ACCAGATCAGGAGCCAACTG |
| LIF_201_rev | CGACTATGCGGTACAGCTCC |
| LIF_fwd | GCTGTACCGCATAGTCGTGT |
| LIF_rev | CCGTAGGTCACGTCCACAT |
| KRT1_fwd | GAAGTCTCGAGAAAGGGAGCA |
| KRT1_rev | ATGGGTTCTAGTGGAGGTATCTA |
| KRT10_fwd | GCAAATTGAGAGCCTGACTG |
| KRT10_rev | CAGTGGACACATTTCGAAGG |
| FLG_fwd | AAAGAGCTGAAGGAACTTCTGG |
| FLG_rev | AACCATATCTGGGTCATCTGG |
| LOR_fwd | CTCACCCTTCCTGGTGCTTT |
| LOR_rev | GGGTGGGCTGCTTTTTCTGA |
| RPL32_fwd | AGGCATTGACAACAGGGTTC |
| RPL32_rev | GTTGCACATCAGCAGCACTT |
