# Supplementary material for: Comprehensive secretome profiling and CRISPR screen identifies SFRP1 as a key inhibitor of epidermal progenitor proliferation
Source: Cell Death Dis. 2025 May 3;16(1):360. doi: 10.1038/s41419-025-07691-0 (PMC12049499; doi:10.1038/s41419-025-07691-0)
Supplement: Supplementary file 11 — Supplemental Table 4 [file 41419_2025_7691_MOESM11_ESM.docx]

**Supplementary Table 4. ATAC PCR primer list.**

| **Name** | **Primer sequences (5’ > 3’)** |
| --- | --- |
| Ad1_noMX | AATGATACGGCGACCACCGAGATCTACACTCGTCGGCAGCGTCAGATGTG |
| Ad2.5 | CAAGCAGAAGACGGCATACGAGATAGGAGTCCGTCTCGTGGGCTCGGAGATGT |
| Ad2.6 | CAAGCAGAAGACGGCATACGAGATCATGCCTAGTCTCGTGGGCTCGGAGATGT |
| Ad2.7 | CAAGCAGAAGACGGCATACGAGATGTAGAGAGGTCTCGTGGGCTCGGAGATGT |
| Ad2.8 | CAAGCAGAAGACGGCATACGAGATCCTCTCTGGTCTCGTGGGCTCGGAGATGT |
