# Supplementary material for: Comprehensive secretome profiling and CRISPR screen identifies SFRP1 as a key inhibitor of epidermal progenitor proliferation
Source: Cell Death Dis. 2025 May 3;16(1):360. doi: 10.1038/s41419-025-07691-0 (PMC12049499; doi:10.1038/s41419-025-07691-0)
Supplement: Supplementary file 12 — Supplemental Table 5 [file 41419_2025_7691_MOESM12_ESM.docx]

**Supplementary Table 5. Stem cell regulation related GO terms for Figure 5E.**

| **Gene** | **GO term** | | **References** |
| --- | --- | --- | --- |
| LIF | GO:0048863 | stem cell differentiation | [PMID:22949634](https://www.ncbi.nlm.nih.gov/entrez/query.fcgi?cmd=Retrieve&db=PubMed&list_uids=22949634&dopt=Abstract) |
| PIM1 | GO:0072091 | regulation of stem cell proliferation | [PMID:23495171](https://www.ncbi.nlm.nih.gov/entrez/query.fcgi?cmd=Retrieve&db=PubMed&list_uids=23495171&dopt=Abstract) |
| WNT7A | GO:0035019 | somatic stem cell population maintenance | [PMID:19497282](https://www.ncbi.nlm.nih.gov/entrez/query.fcgi?cmd=Retrieve&db=PubMed&list_uids=19497282&dopt=Abstract) |
| OVOL1 | GO:2000647 | negative regulation of stem cell proliferation | [PMID:24735878](https://www.ncbi.nlm.nih.gov/entrez/query.fcgi?cmd=Retrieve&db=PubMed&list_uids=24735878&dopt=Abstract) |
|  | GO:0072091 | regulation of stem cell proliferation | [PMID:24735878](https://www.ncbi.nlm.nih.gov/entrez/query.fcgi?cmd=Retrieve&db=PubMed&list_uids=24735878&dopt=Abstract) |
| SHC4 | GO:0048863 | stem cell differentiation | [PMID:22948967](https://www.ncbi.nlm.nih.gov/entrez/query.fcgi?cmd=Retrieve&db=PubMed&list_uids=22948967&dopt=Abstract) |
| SFRP1 | GO:0048865 | stem cell fate commitment | [PMID:20130188](https://www.ncbi.nlm.nih.gov/entrez/query.fcgi?cmd=Retrieve&db=PubMed&list_uids=20130188&dopt=Abstract) |
|  | GO:0048863 | stem cell differentiation | [PMID:20130188](https://www.ncbi.nlm.nih.gov/entrez/query.fcgi?cmd=Retrieve&db=PubMed&list_uids=20130188&dopt=Abstract) |
|  | GO:0035019 | somatic stem cell population maintenance | [PMID:19664990](https://www.ncbi.nlm.nih.gov/entrez/query.fcgi?cmd=Retrieve&db=PubMed&list_uids=19664990&dopt=Abstract) |
