# Supplementary material for: Comprehensive secretome profiling and CRISPR screen identifies SFRP1 as a key inhibitor of epidermal progenitor proliferation
Source: Cell Death Dis. 2025 May 3;16(1):360. doi: 10.1038/s41419-025-07691-0 (PMC12049499; doi:10.1038/s41419-025-07691-0)
Supplement: Supplementary file 13 — Supplemental Table 6 [file 41419_2025_7691_MOESM13_ESM.docx]

**Supplementary Table 6. Stem cell regulation related GO terms for Supplementary Figure 4 D, E.**

| GO:0005020 | stem cell factor receptor activity |
| --- | --- |
| GO:0005173 | stem cell factor receptor binding |
| GO:0017145 | stem cell division |
| GO:0019827 | stem cell population maintenance |
| GO:0035019 | somatic stem cell population maintenance |
| GO:0035701 | hematopoietic stem cell migration |
| GO:0036335 | intestinal stem cell homeostasis |
| GO:0048103 | somatic stem cell division |
| GO:0048863 | stem cell differentiation |
| GO:0048864 | stem cell development |
| GO:0048865 | stem cell fate commitment |
| GO:0060218 | hematopoietic stem cell differentiation |
| GO:0060529 | squamous basal epithelial stem cell differentiation involved in prostate gland acinus development |
| GO:0072091 | regulation of stem cell proliferation |
| GO:0097150 | neuronal stem cell population maintenance |
| GO:1902033 | regulation of hematopoietic stem cell proliferation |
| GO:1902459 | positive regulation of stem cell population maintenance |
| GO:1904672 | regulation of somatic stem cell population maintenance |
| GO:1904674 | positive regulation of somatic stem cell population maintenance |
| GO:1904675 | regulation of somatic stem cell division |
| GO:1904677 | positive regulation of somatic stem cell division |
| GO:2000035 | regulation of stem cell division |
| GO:2000036 | regulation of stem cell population maintenance |
| GO:2000101 | regulation of mammary stem cell proliferation |
| GO:2000103 | positive regulation of mammary stem cell proliferation |
| GO:2000647 | negative regulation of stem cell proliferation |
| GO:2000736 | regulation of stem cell differentiation |
| GO:2000737 | negative regulation of stem cell differentiation |
| GO:2000738 | positive regulation of stem cell differentiation |
| GO:2000739 | regulation of mesenchymal stem cell differentiation |
| GO:2000741 | positive regulation of mesenchymal stem cell differentiation |
